# Photon-assisted tunnelling with nonclassical light

**DOI:** 10.1038/ncomms6562

**Published:** 2014-11-26

**Authors:** J. -R. Souquet, M. J. Woolley, J. Gabelli, P. Simon, A. A. Clerk

**Affiliations:** 1 Laboratoire de Physique des Solides, Université Paris-Sud, Orsay 91405, France; 2 Department of Physics, McGill University, Montréal, Quebec, Canada; 3 School of Engineering and Information Technology, University of New South Wales, ADFA, Canberra, Australian Capital Territory 2600, Australia

## Abstract

Among the most exciting recent advances in the field of superconducting quantum circuits is the ability to coherently couple microwave photons in low-loss cavities to quantum electronic conductors. These hybrid quantum systems hold great promise for quantum information-processing applications; even more strikingly, they enable exploration of new physical regimes. Here we study theoretically the new physics emerging when a quantum electronic conductor is exposed to nonclassical microwaves (for example, squeezed states, Fock states). We study this interplay in the experimentally relevant situation where a superconducting microwave cavity is coupled to a conductor in the tunnelling regime. We find that the conductor acts as a nontrivial probe of the microwave state: the emission and absorption of photons by the conductor is characterized by a nonpositive definite quasi-probability distribution, which is related to the Glauber–Sudarshan *P*-function of quantum optics. These negative quasi-probabilities have a direct influence on the conductance of the conductor.

## Abstract (web summary)

Coherently coupling microwave photons to quantum electronic conductors could provide a useful platform for quantum information processing. Souquet *et al*. now theoretically demonstrate that such systems can also act as sensitive probes of the quantum properties of non-classical microwave radiation.

## Graphical abstract

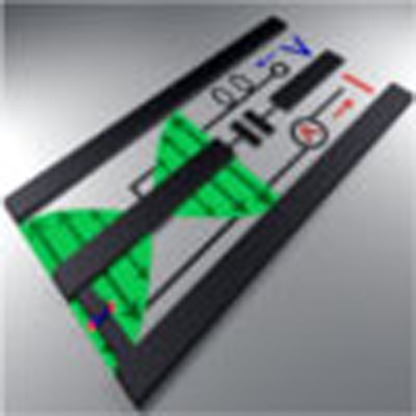

The physics of a tunnel junction illuminated by a purely classical microwave field has been understood since the 1960s with the classic work of Tien and Gordon^1^. This situation is equivalent to simply having an ac bias voltage across the conductor, and the resulting modification of the current is known as photon-assisted tunnelling; it has been measured in countless experiments (for example, refs 2, 3, 4). Despite the word ‘photon’ in the effect’s name, in this standard formulation there is nothing quantum in the treatment of the applied microwave field.

To study a more truly quantum version of photon-assisted tunnelling, one could consider driving a tunnel junction with a quantum microwave field produced in a cavity. The cavity effectively acts as an ac voltage bias across the conductor; by maintaining the cavity in a nonclassical state, the junction is exposed to a nontrivial microwave field. Our goal will be to understand how such nonclassical microwaves affect electronic transport. Such cavity-plus-conductor set-ups have been realized experimentally, both in experiments using metallic tunnel junctions^5^,^6^,^7^, as well as more recent experiments with high-*Q* microwave cavities coupled to either quantum dots^8^,^9^ or carbon nanotubes^10^. Note that the converse problem of how an electronic conductor can be used to produce nonclassical squeezed microwaves was recently studied experimentally^11^. Motivated by experiments with superconducting circuits, theoretical work has also studied transport in undriven dot-plus-cavity set-ups^12^,^13^,^14^,^15^.

If the cavity is not driven (that is, not coherently populated with photons), the cavity-plus-conductor set-up realizes another well-studied quantum transport problem: dynamical Coulomb blockade (DCB)^16^,^17^,^18^,^19^. Here the cavity acts as a structured electromagnetic environment for the junction, one that can absorb (and at non-zero temperature, emit) energy from tunnelling electrons. The standard theory of this effect^18^,^19^,^20^ is based on the function *P*(*E*), which gives the probability of the environment absorbing an energy *E* from a tunnelling electron. DCB has been experimentally probed both for nonresonant environments^21^,^22^,^23^,^24^,^25^ as well as for environments formed by resonators^5^,^6^,^7^, with excellent theoretical agreement. In stark contrast to standard DCB, our focus will be on a nonequilibrium environment produced by preparing a cavity in a nonclassical state.

In this paper, we develop a comprehensive theory describing how nonequilibrium, driven states of a microwave cavity influence electronic transport in a coupled tunnel junction, with a particular focus on cavities which are maintained in truly nonclassical states (such as a Fock state or a squeezed state). Generalizing both standard photon-assisted tunnelling theory and dynamical Coulomb blocakde theory, we show that the emission and absorption of photons by the conductor is naturally characterized by a quasi-probability distribution, which can fail to be positive. The resulting negative probabilities can have a direct influence on both the conductance and finite-frequency current noise of the tunnel junction. We also show that this new quasi-probability distribution has a direct connection to the well-known Glauber–Sudarshan *P*-function of quantum optics. We present results for parameter regimes relevant to state-of-the-art experiments, and show that for sufficiently large tunnel resistances, the tunnel junction acts as a nontrivial and nonlinear probe of the cavity state.

## Results

### Model

**Figure 1:**
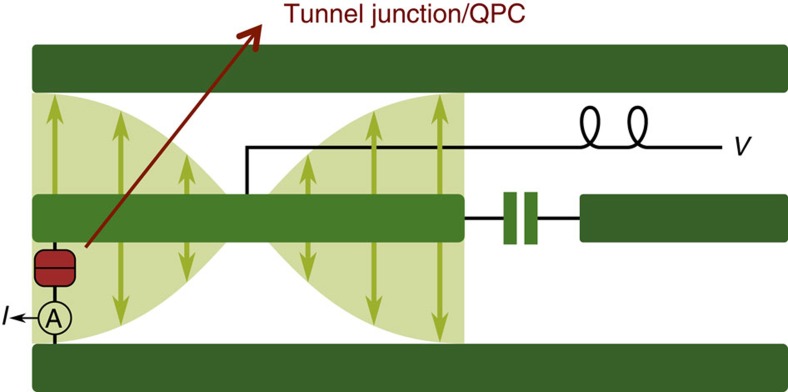
Proposed set-up. Schematic showing a resonant mode of a half-wavelength coplanar waveguide resonator, with a quantum conductor (tunnel junction or quantum point contact (QPC)) that contacts the centre strip and lower ground plane at a voltage antinode. A dc voltage is also applied to the junction via the centre strip at a voltage node, so as not to induce losses (see, for example, refs 26, 27, 28). The state of the resonant mode provides a quantum ac voltage across the junction; we are interested in how this influences the dc junction current *I* and what this current reveals about the quantum voltage.

We consider transport through a voltage-biased tunnel junction (dc bias voltage *V*), which is coupled to the voltage antinode of a microwave cavity in such a way that the cavity voltage acts as an additional bias voltage across the junction. One possible realization is depicted in Fig. 1, where a tunnel junction is coupled to a coplanar waveguide resonator (see also refs 26, 27, 28). We calculate the average current to lowest nonvanishing order in the tunnelling strength. If the resonator was in thermal equilibrium, we would recover the standard DCB expression^20^. We generalize this approach to now allow for an environment (that is, the cavity), which is in an *arbitrary* nonequilibrium, non-stationary state. In general, the average tunnel current is time-dependent and can be written as


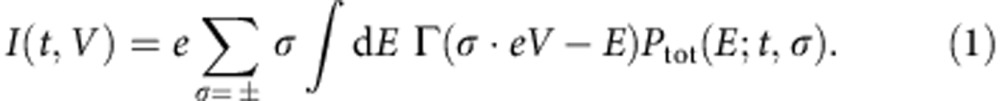


The two terms here represent (respectively) left-to-right and right-to-left tunnelling, and Γ(*E*) describes the energy-dependent tunnelling rate of the uncoupled junction. For the usual case of metallic leads, one has Γ(*E*)=(*e*^2^*R*_T_)^−1^*E*/(1−exp(−*E*/*k*_B_*T*_el_)), where *T*_el_ is the lead temperature and *R*_T_ is the junction resistance (see, for example, ref. 29). In this standard case, the current of the uncoupled junction is purely Ohmic, *I*_0_(*V*)=*V*/*R*_T_. The functions *P*_tot_(*E*;*t*,*σ*) describe energy transfer to/from the electromagnetic environment. They are given by a causal environment correlation function, evaluated in the absence of tunnelling (see Supplementary Note 1 for a full derivation):


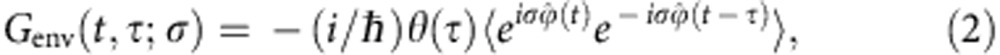



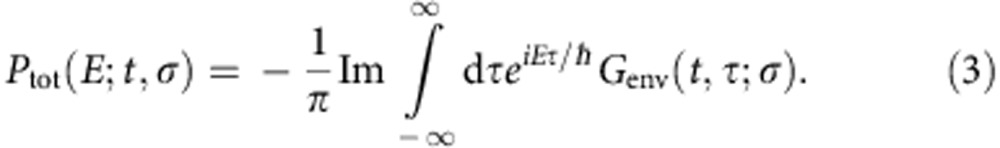


Here 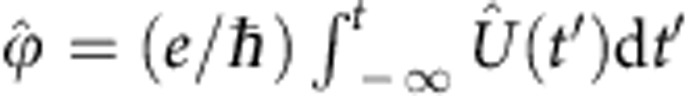 is the phase operator, defined in terms of the (Heisenberg picture) environment voltage operator 
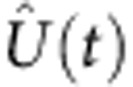
. As shown in Supplementary Note 1, equations (1)–(3), 2, 3 reduce to standard DCB expressions in the usual case of a thermal environment; equation (3) yields a *P*_tot_(*E*) function, which is positive definite and only depends on *E*.

In our system, we treat the environment as a single resonant mode of a cavity, which can be represented as a quantum *LC* circuit with frequency Ω=1/(*LC*)^1/2^. 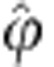 is thus given by one quadrature of the cavity mode annihilation operator 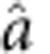 (ref. 30),


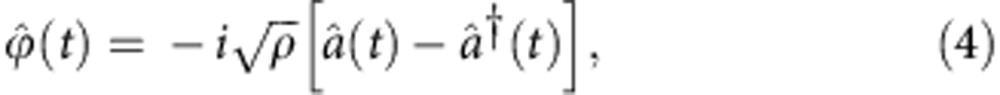


with *ρ*=*πZ*_cav_/*R*_K_ parameterizing the strength of zero-point voltage fluctuations in the cavity (
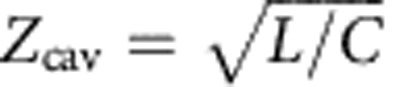
, *R*_K_=*h*/*e*^2^ the resistance quantum). As we will see, the most interesting regime in our system is when 
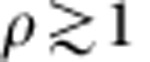
. Using only geometric inductances, *Z*_cav_ is limited by the vacuum impedance^31^, implying 
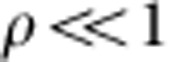
. However, much larger impedances are possible using Josephson junction arrays. Recent experiments have achieved high-*Q* microwave cavities that have large inductances (~100 nH), implying *ρ*~1 (ref. 32). The recent work of ref. 33 even studied transport in a tunnel junction coupled to a high-impedance resonator, achieving *ρ*~0.3. The rapid progress here suggests that even larger values of *ρ* should be possible in the near term.

Our focus will be on situations where the cavity is maintained in some interesting nonvacuum state, either by continuous driving, or via reservoir-engineering techniques^34^, which have been used in several recent circuit QED experiments^35^,^36^. In either case, this involves coupling the cavity to an external dissipative channel; this gives the cavity a finite damping rate *κ*. Our main goal in what follows is to understand how transport through the tunnel junction can be used as a probe of the (possibly nonclassical) cavity state. We thus ideally would like the backaction disturbance of the cavity state by the conductor to be minimal. For clarity, we will thus focus our discussion on regimes where this is the case. Formally, the backaction-induced modification of the cavity state does not contribute to the junction current to leading order in the tunnelling; neglecting it is thus consistent with our perturbative treatment. In the Methods section, we consider the impact of non-zero backaction and show that it can be neglected if the tunnel resistance of the junction *R*_T_ is sufficiently large. As discussed in the Methods section, for 
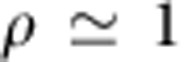
 and *κ*~10^−2^Ω, one needs 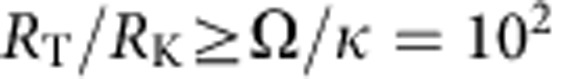. Such values can be obtained by using a sufficiently small and opaque metallic tunnel junction or by using a single channel quantum point contact in the tunnel regime (see for example, ref. 25). We stress that, while our approach treats the electron tunnelling perturbatively, the coupling between the cavity and conductor (parameterized by *ρ*) is not assumed to be small.

Finally, for the conductor to accurately probe the cavity state at the single photon level, one ideally also wants the electronic temperature *T*_el_ to satisfy 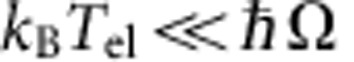. As typical experiments involve GHz-frequency resonators and mK temperatures, this condition is well satisfied, implying that the role of thermal fluctuations (both in the cavity and in the electronic reservoirs) will be minimal. For clarity, we will thus focus on the zero-temperature limit in what follows; the small effect of non-zero temperature is discussed in the Methods section.

### Closed cavity

The simplest situation to consider is where the coupling *κ* to the dissipative channel used to maintain the cavity state is strong enough to maintain the cavity in the desired state irrespective of the junction current, but still weak enough that it does not appreciably modify the cavity dynamics. We start by analysing this situation, meaning that we can neglect the effects of *κ* in calculating *P*_tot_(*E*;*t*,*σ*); non-zero *κ* will be addressed in the next section.

In general, one finds that *P*_tot_(*E*;*t*,*σ*) and hence the average current oscillates as a function of *t*. We will focus on the dc current, and thus average over *t*. The resulting *P*_tot_(*E*) function is then only a function of *E*. In the *κ*→0 limit, the energy of a cavity photon is precisely *ℏ*Ω, and hence *P*_tot_(*E*) has the form


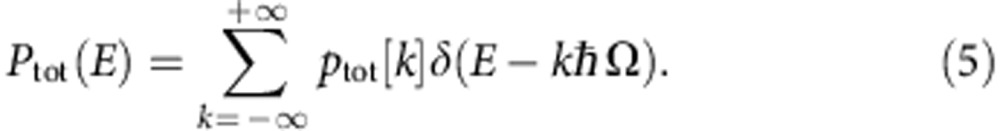


For a simple Ohmic tunnel junction, the differential dc conductance d*I*/d*V* will then exhibit a series of steps as a function of dc voltage *V*, as different photon-assisted processes become energetically allowed. As discussed in the Methods section, by measuring d*I*/d*V* and the (symmetrized) finite-frequency junction current noise 
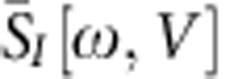
, one can directly extract the weights *p*_tot_[*k*].

Without dissipation, the cavity evolves freely, and we can calculate *P*_tot_(*E*) for an arbitrary cavity state 
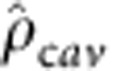
. It can be written as the convolution of two normalized distributions,


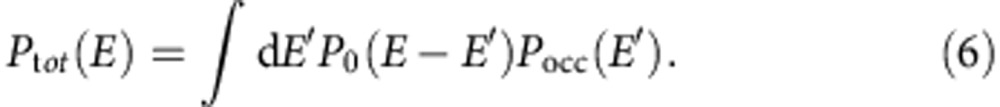


*P*_0_(*E*) describes the absorption of energy by a ground-state cavity and only has weight for *E*≥0. In contrast, *P*_occ_(*E*) is a quasi-probability distribution that describes the additional emission and absorption processes possible when the cavity is occupied with photons. If the cavity was in its ground state, we would simply have *P*_occ_(*E*)=*δ*(*E*) and *P*_tot_(*E*)=*P*_0_(*E*). *P*_0_(*E*) is a Poisson distribution with mean *ρ* (refs 18, 20):


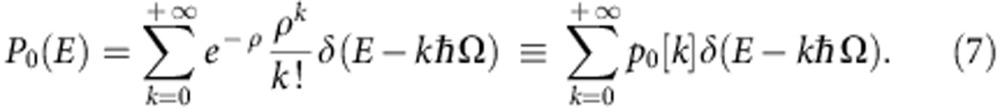


The function *P*_occ_(*E*) that we introduce captures the novel physics we are after. For an arbitrary cavity state 
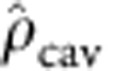
, it is directly related to the Glauber–Sudarshan *P*-function 
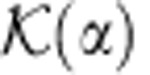
, which represents 
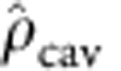
 via a quasi-probability distribution in phase space. Recall that 
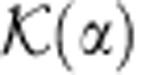
 is defined via^37^


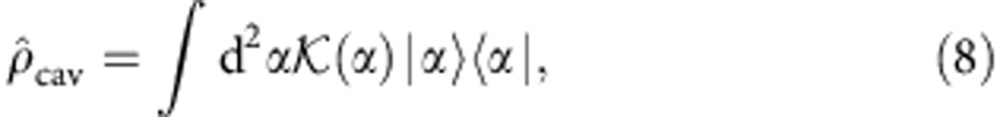


where |*α*› denotes a cavity coherent state with complex amplitude *α*. 
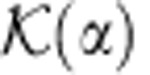
 expresses 
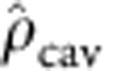
 as an incoherent mixture of coherent states.

For *κ*→0, we find that *P*_occ_(*E*) also reduces to a discrete distribution,


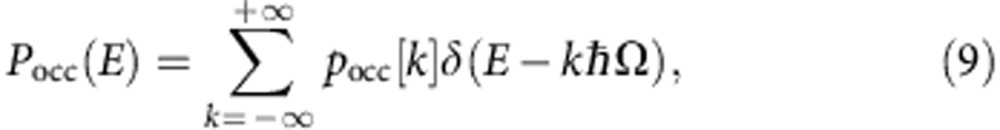


with weights directly determined by the Glauber–Sudarshan *P*-function:


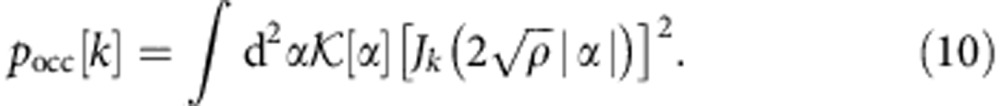


Here *J*_*k*_ is a Bessel function.

If *P*_occ_(*E*) is positive definite, equation (6) implies that we can interpret the energy *E* absorbed by the cavity in a tunnel event as the sum of two independent stochastic quantities: an amount associated with vacuum fluctuations (as described by *P*_0_) and an amount associated with the population of the cavity (as described by *P*_occ_). While *P*_tot_(*E*) must always be positive definite (see Supplementary Note 1), this is not necessarily true of *P*_occ_(*E*): it can become negative for nonclassical cavity states, that is, states whose phase-space distribution 
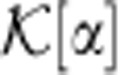
 either fails to be positive definite or is highly singular^37^. Negativity in *P*_occ_(*E*) will thus be a direct sign of nonclassical light.

For further intuition into equation (10), consider the simple case where the cavity is in a coherent state with amplitude 
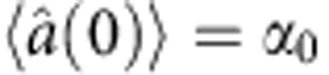
. In this case 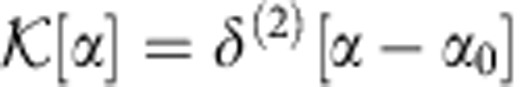, and


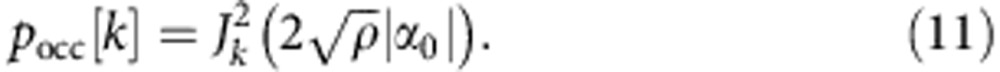


*p*_occ_[*k*] is precisely the weight for an *k*-photon process in the standard Tien–Gordon theory for a purely classical ac voltage *V*_ac_(*t*)∝|*α*_0_| (ref. 1). Thus, equation (10) demonstrates that for a general state, *p*_occ_[*k*] is a sum of Tien–Gordon distributions for different amplitudes, with each term weighted by the Glauber–Sudarshan P-function 
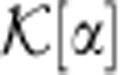
.

Returning to the coherent state case, we see from equation (6) that the full distribution *P*_tot_(*E*) involves convolving the Tien–Gordon distribution with the zero-temperature absorption processes of the cavity. This thus generalizes Tien–Gordon theory to include the contribution of cavity vacuum noise. Note that a purely classical ac voltage does not modify the dc *I*–*V* characteristic of a conventional tunnel junction because of the lack of any rectification (that is, such a junction has a purely linear *I*–*V* characteristic). This is however no longer true when we include zero-point fluctuations of the field: now, the dc *I*–*V* characteristic of the junction is indeed modified by the presence of the ac voltage. This behaviour is demonstrated in Fig. 4.

### Fock state

Consider now the case where the cavity is stabilized in a Fock state |*n*›; this has been achieved recently via reservoir-engineering protocols in circuit QED^38^. For the simple case *n*=1, one finds *p*_occ_[*k*]=0 unless *m*=0, ±1, in which case:


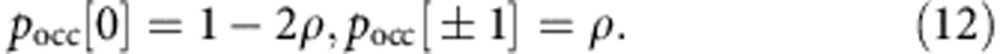


**Figure 2:**
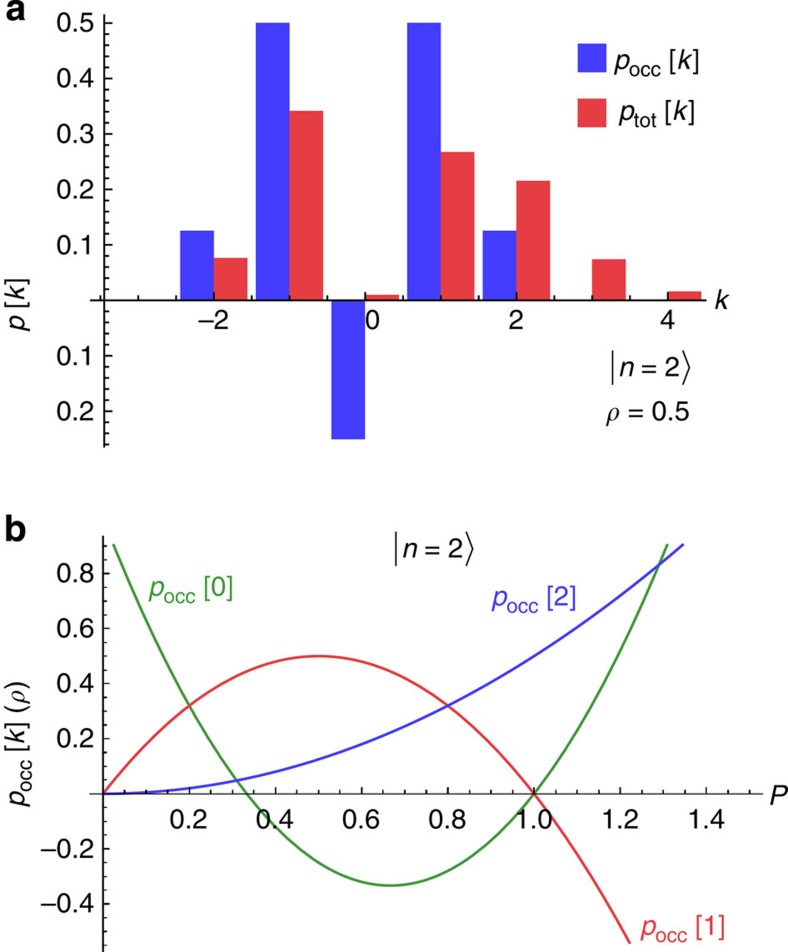
Quasi-probabilities for two-photon Fock state. (**a**) Probability distributions describing photon emission and absorption by a cavity initially prepared in the *n*=2 Fock state, in the absence of cavity damping, and for a dimensionless cavity impedance *ρ*≡*πZ*_cav_/*R*_K_=0.5. The quasi-probabilities *p*_occ_[*k*] characterize the additional photon emission/absorption processes possible because of populating the cavity with photons, whereas the probabilities *p*_tot_[*k*] also include the absorption events associated with vacuum noise. While *p*_tot_[*k*] must always be positive definite, *p*_occ_[*k*] can fail to be positive for nonclassical cavity states. Here we see that the weight *p*_occ_[*k*=0]≤0. (**b**) Behaviour of the quasi-probabilities *p*_occ_[*k*] for a closed cavity in the *n*=2 Fock state, as a function of *ρ* (which characterizes the strength of cavity zero-point voltage fluctuations seen by the conductor). Negativity requires sufficiently large *ρ*, although the minimum required *ρ* decreases with increasing *n*.

*P*_occ_(*E*) for this state describes the possibility to emit or absorb 0 or 1 photons because of the non-zero cavity population. The quasi-probability for the 0-photon process becomes negative for *ρ*>1/2. Similar negativity is found for other Fock states (see Supplementary Note 2 and Fig. 2); the larger the value of *n*, the smaller the value of *ρ* needed to see negativity. As discussed, this negativity is a direct consequence of the nonclassical nature of the cavity state.

**Figure 3:**
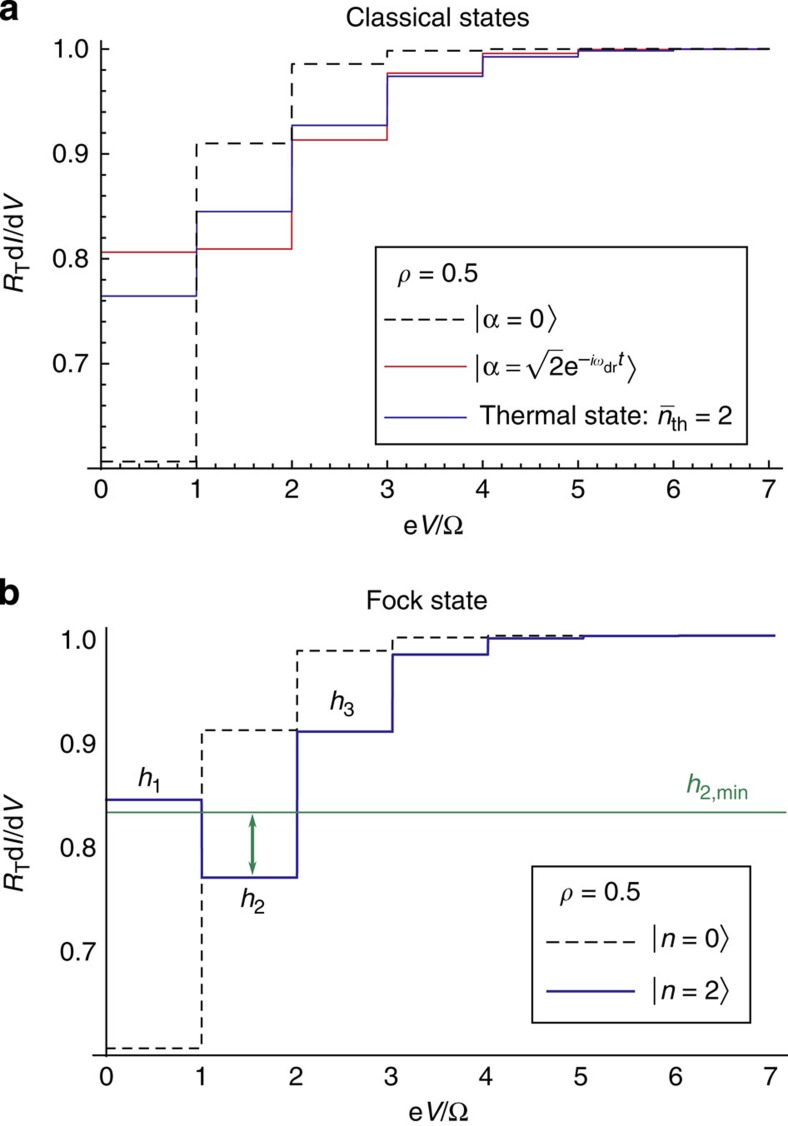
Differential conductance for classical states versus Fock states. (**a**) Differential conductance d*I*/d*V* versus dc bias voltage *V* for a tunnel junction coupled to a cavity having dimensionless impedance *ρ*=0.5. We assume that the cavity is initially prepared in some specific state and neglect cavity dissipation for simplicity; we also take the limit of a negligible electron temperature, 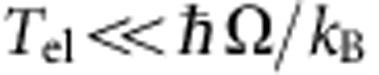. The dashed curve is for a ground-state cavity, the solid blue curve for a thermal state with an average photon number 
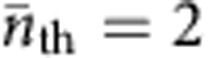
, and the red curve for a coherent state with average photon number |*α*|^2^=2. For a thermal cavity state, the conductance plateaus are always monotonically increasing in height with *V*. (**b**) Same as **a**, but now the solid curve corresponds to a cavity prepared in the Fock state |*n*=2›. The striking signature of a nonclassical state here is the strongly non-monotonic dependence of the first few conductance plateaus on voltage; in particular, the height of the second plateau (*h*_2_) is smaller than the first (*h*_1_). This is in sharp contrast to the classical states shown in **a**, states which all have an identical average cavity photon number. As discussed in Supplementary Note 3, if one assumes that the distribution *P*_occ_(*E*) describing the cavity is positive definite, then one can rigorously bound how small *h*_2_ can be relative to the average height of the first and third plateaus. This bound is shown as the green horizontal line in the figure. The conductance clearly violates this bound, and thus provides direct (and experimentally accessible) evidence for the negativity in *P*_occ_(*E*). Similar violations are possible with other choices of Fock state; higher *n* Fock states allow violations at even smaller values of *ρ* (see Supplementary Note 2).

The negativity in *P*_occ_(*E*) leads to a distinct signature in the differential dc conductance of the junction (see Fig. 3). The conductance exhibits regular plateaus as a function of dc voltage. However, unlike the case of a cavity thermal state, the plateau heights associated with a cavity Fock state do not increase monotonically with voltage. These surprising decreases in conductance plateau height are inconsistent with *P*_occ_(*E*) being positive definite. As shown in the Methods section, if *P*_occ_(*E*) were positive, there is a bound on how small the second plateau in d*I*/d*V* can be compared with the first and third plateaus. This bound is generically violated by the d*I*/d*V* obtained with a Fock state in the cavity (for example, that shown in Fig. 3). Thus, the differential conductance of the junction provides a direct probe of the nonclassical nature of the cavity state.

Further evidence of the negativity in the Fock state *p*_occ_[*k*] can be seen in the corresponding total emission/absorption probability *p*_tot_[*k*] (which includes the contribution from vacuum noise). For a cavity maintained in an *n*-photon Fock state, we find (see Supplementary Note 2):


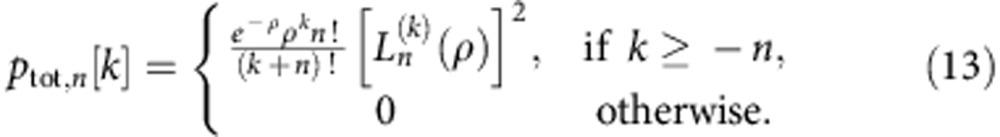


Here 
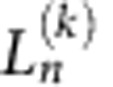
 denotes a generalized Laguerre polynomial. As expected, if the cavity is maintained in an *n*-photon Fock state, then in a single tunnel event at most *n* photons can be absorbed. However, for an appropriately chosen *ρ*, *p*_tot,n_[−*k*] can be zero for *k*<*n*, while at the same time *p*_tot_[−(*k*+1)] is non-zero. Such a cancellation would be impossible if *p*_occ_[*k*] were positive definite: if the probability to absorb *k*+1 photons from the junction is non-zero, then the probability to absorb *k* photons must also be non-zero. This is a simple consequence of *p*_tot_[*k*] being the convolution of *p*_occ_[*k*] with a Poisson distribution, *p*_0_[*k*].

As discussed in the Methods section, one can directly measure *p*_tot_[*k*] if one measures both the dc conductance of the junction and its finite-frequency current noise. Using such a measurement to detect the vanishing of *p*_tot,n_[−*k*] for *k*≤*n* would thus also provide direct evidence for the nonclassical nature of the cavity state. If one knows *p*_tot_[*k*], one can also undo the convolution in equation (6) and extract the (possibly negative) quasi-probability distribution *p*_occ_[*k*]. Writing things explicitly, we have:


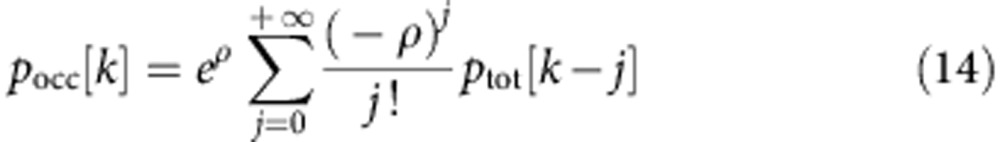


### Cavity driving and dissipation

We now consider the case where the cavity is maintained in an interesting state via continuous driving through an input port, including the non-zero cavity dissipation associated with this port. Our approach extends easily to such situations if the driving field is Gaussian; this includes the interesting case of a squeezed vacuum state input. Letting *κ* denote the damping rate because of the coupling to the transmission line used to drive the cavity, one can use standard input–output theory^30^ to derive a Heisenberg–Langevin equation for the cavity field (see Methods). For Gaussian states, this equation can be solved to obtain the phase–phase correlator and hence *P*_tot_(*E*).

We find that even for a driven, dissipative cavity, *P*_tot_(*E*) can still be written in the general form of equation (6). The distribution *P*_0_(*E*) describes photon absorption by the cavity when it is driven solely by vacuum noise:


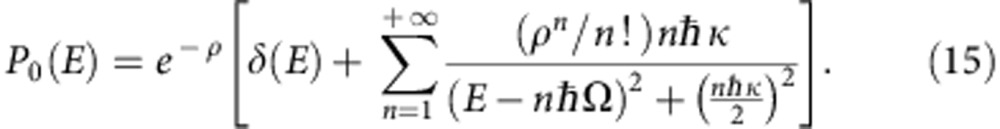


In comparison with equation (7), the effects of dissipation are to simply broaden the peaks associated with absorbing *n*≥1 photons. The distribution *P*_occ_(*E*) again describes additional absorption/emission processes possible when the cavity drive populates the cavity.

**Figure 4:**
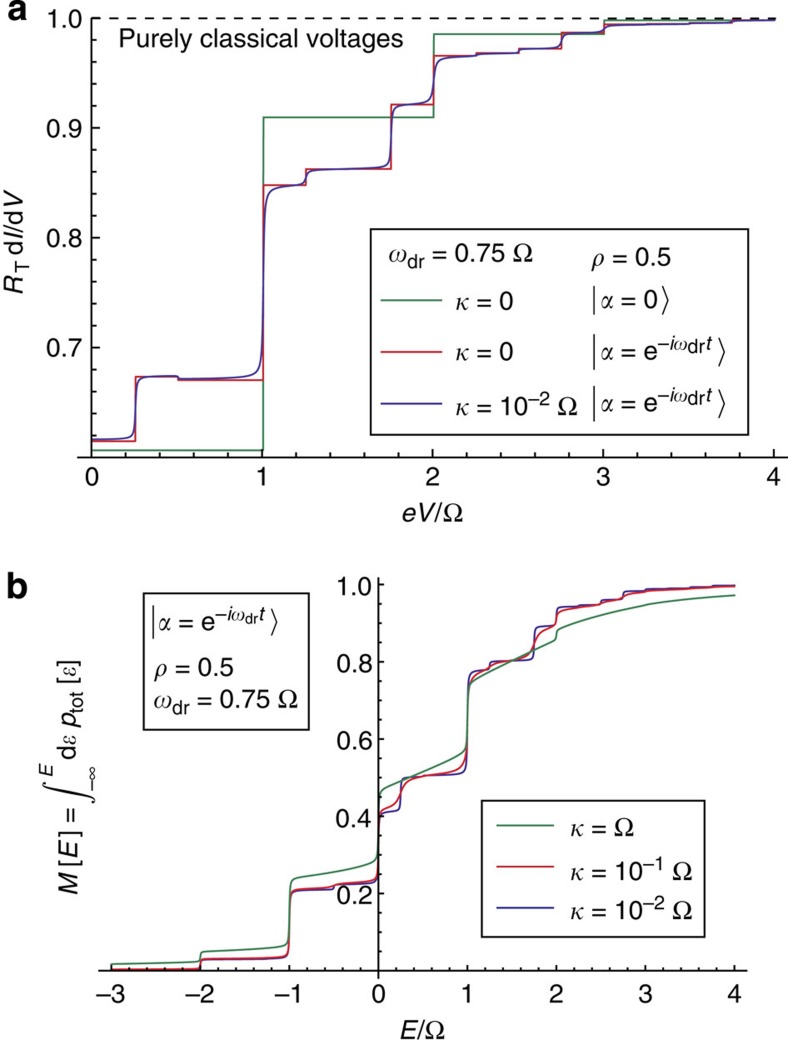
Results for a damped cavity driven with coherent state driving. (**a**) Differential conductance d*I*/d*V* versus dc bias voltage *V* for a tunnel junction coupled to a cavity that is continuously driven into a coherent state having amplitude |*α*|=1. We have taken a drive frequency that is detuned from resonance: *ω*_dr_=0.75Ω. Results for zero dissipation (*κ*→0) and finite dissipation *κ*=0.01Ω are shown; all curves correspond to zero cavity and electron temperature (see Methods for finite temperature effects). The steps in the conductance now occur at multiples of both the cavity and the drive frequency. Note that standard photon-assisted tunnelling theory (Tien–Gordon theory^1^) predicts that d*I*/d*V*=1/*R*_T_ independent of *V* and the ac voltage. Classically, this is because of the linear *I*–*V* characteristic of a tunnel junction and consequent lack of any rectification. The behaviour shown here is starkly different because of the inclusion of zero-point fluctuations. (**b**) Integrated probability function *P*_tot_(*E*) for the same situation as **a**. One again sees steps at multiples of the the cavity resonance frequency and at multiples of the drive frequency. The steps associated with the drive frequency remain sharp even in the presence of cavity dissipation.

For the coherent state case, we take the cavity to be driven at a frequency *ω*_dr_; in this case the average cavity amplitude is 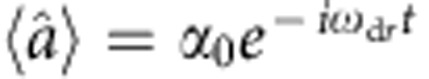. We find that *P*_occ_(*E*) is again given by the closed-cavity expression equations (9)–(11), 28, 32, except that one replaces the cavity frequency Ω with the drive frequency *ω*_dr_. In contrast to the vacuum absorption peaks, these processes are not lifetime broadened and correspond to a photon frequency set by the drive frequency *ω*_dr_, and not the cavity resonance frequency Ω. Both these features lead to interesting signatures in the differential conductance; in particular, one sees steps in the conductance corresponding to both relevant photon frequencies (the drive frequency and the cavity resonance frequency). This behaviour is demonstrated in Fig. 4.

### Squeezed state

Consider next a cavity that is maintained in a squeezed state, where the variance of one quadrature is reduced below the zero-point value by a factor *e*^−2*r*^ (*r*>0). While such a state is Gaussian, it yields a highly singular Glauber–Sudarshan *P*-function and is thus considered to be nonclassical^37^. A squeezed state could be maintained in a superconducting cavity using reservoir-engineering techniques^39^. Alternatively, one could simply drive the cavity with squeezed vacuum noise (as produced by a parametric amplifier); this kind of intracavity squeezing has been recently realized in experiment^40^. We focus on this situation in what follows.

**Figure 5:**
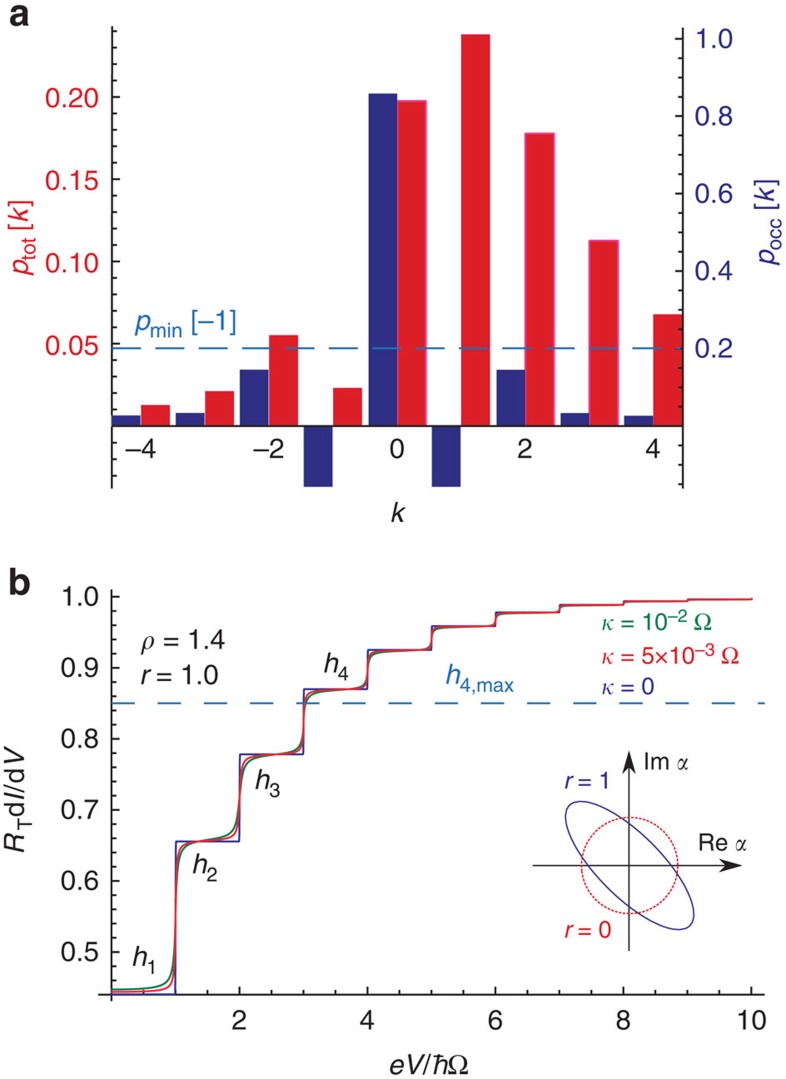
Results for cavity-squeezed states. (**a**) Probability distributions *p*_tot_[*k*] and *p*_occ_[*k*] associated with a dissipation-free cavity in a squeezed vacuum state, with *ρ*=1.4. We take the squeeze parameter to be *r*=1, meaning that the variance of one cavity quadrature is reduced to 1/*e*^2^~0.14 of its ground state value ground state (see inset of **b**). *p*_occ_[*k*] describes the extra photon absorption and emission processes possible when the cavity is occupied with photons, whereas the distribution *p*_tot_[*k*] also includes the additional absorption processes associated with vacuum fluctuations. Similar to a Fock state, the squeezed-state quasi-probabilities *p*_occ_[*k*] can exhibit negativity, which occurs here most strongly for *k*=±1. This in turn leads to a strong suppression in the value of the distribution *p*_tot_[*k*] at *k*=−1. If *p*_tot_[*k*] were positive, then *p*_tot_[−1] has a minimum possible value *p*_min_[−1] (dashed green line); this lower bound is based on the values of *p*_tot_[*k*] at *k*=−2,−3. As clearly shown in the figure, the negativity in *p*_occ_[±1] causes a large violation of this bound. (**b**) Differential conductance for the the same set-up in **a**; we now, however, also include the effects of non-zero cavity damping *κ*. By measuring the heights of the first three conductance plateaus (*h*_1_−*h*_3_), one can bound the maximum possible value of the fourth plateau (*h*_4_) possible with any positive definite *p*_occ_[*k*]. This value is *h*_4,max_ and is indicated with a horizontal line. We see that the conductance violates this bound, and thus provides direct evidence for the negativity of *p*_occ_[*k*]. One can also directly measure the *p*_tot_[*k*] shown in **a** by combining the conductance measurement shown here with a measurement of the excess current noise 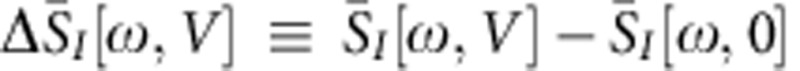 (see Methods). All curves correspond to zero electron and cavity temperatures (see Methods for finite temperature effects).

Analytic expressions can be obtained for *P*_occ_(*E*) in the case of a cavity driven by squeezed microwaves, see Supplementary equation (28). One finds that *P*_occ_(*E*) for 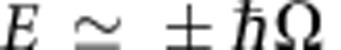 can become negative when 
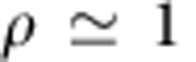
. As discussed in the caption of Fig. 5, this leads to a striking suppression of the peak in *P*_tot_(*E*) near *E*=−*ℏ*Ω, which describes the possibility to absorb a single photon. The weight of this process is suppressed more than would ever be possible if *P*_occ_(*E*) were positive definite. Thus, by measuring *P*_tot_(*E*), for a squeezed state, one could directly infer the negativity of *P*_occ_(*E*).

As shown in Fig. 5, this negativity-induced suppression of *P*_tot_(*E*) yields a direct signature in the conductance: the height of the fourth conductance plateau is higher than would be possible with any positive definite *P*_occ_(*E*). In this figure, we also show results including finite cavity dissipation; for small levels of dissipation (
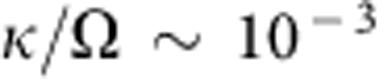
) the results are unchanged. *P*_tot_(*E*) could also be extracted directly if one measures both the differential conductance of the junction and the finite-frequency junction current noise (see Methods). Note that the finite-frequency current noise measurements for quantum point contacts having *R*_T_>>*R*_K_ (as we require here) have been performed previously^41^.

### Conclusion

We have studied the interplay of nonclassical light with electron transport through a tunnel junction, showing that this basic light–matter interaction is naturally characterized by negative quasi-probabilities for truly quantum states. This negativity leads to direct signatures in the differential conductance of the conductor, signatures that should be accessible in state-of-the-art experiments. Our results can directly be generalized to describe biased Josephson junctions interacting with quantum light; such systems allow even larger values of *ρ* (ref. 20). They also suggest the general potential of using quantum conductors as a powerful tool to characterize, and perhaps control, quantum microwave states in hybrid systems incorporating superconducting microwave cavities and semiconductor electronic devices.

## Methods

### Quasi-probability distributions for a closed cavity

Using the definition of the Glauber–Sudarshan *P*-function 
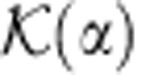
 in equation (8) and the fact that 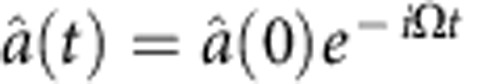 for a closed cavity, one can explicitly calculate the RHS of equation (3). Averaging over the observation time *t* then yields equation (10).

Alternatively, one can express *P*_occ_(*E*) as (*ℏ*=1)


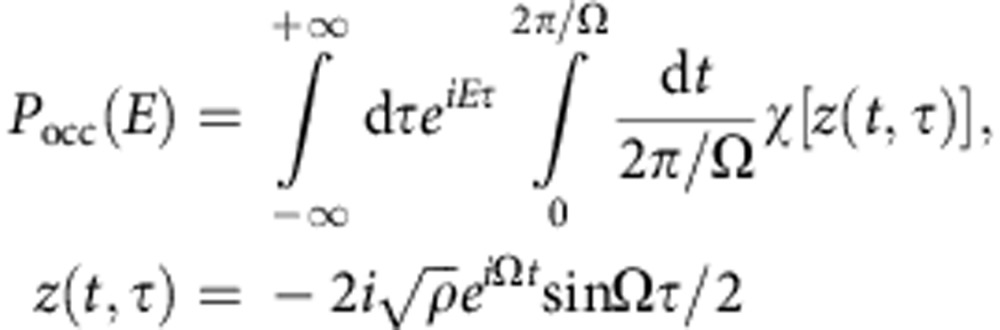


where we have shifted the time argument in equation (2) by *t*→*t*−*τ*/2 for clarity, and where the characteristic function *χ*[*λ*] is defined as


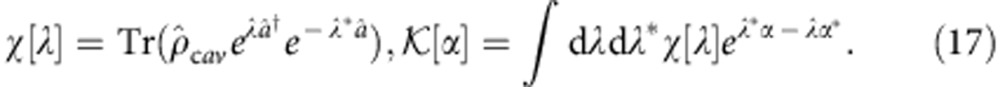


It follows that *P*_occ_(*E*)=*P*_occ_(−*E*), regardless of the cavity state (that is, there is a perfect symmetry between absorption and emission processes, as one might expect for purely classical noise^30^). *P*_occ_(*E*) is determined by the normal-ordered expectations 
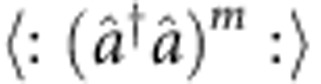
 of the cavity state; the larger the value of *ρ*, the more sensitive one is to higher moments.

### Connecting transport to probabilities

For low electron temperatures 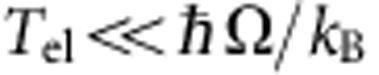, the differential conductance of the junction will exhibit sharp steps as a function of *V*, with transitions at *eV*=*mℏ*Ω; these steps correspond to turning on and off photon-assisted processes. We define the normalized height of the *m*th step as


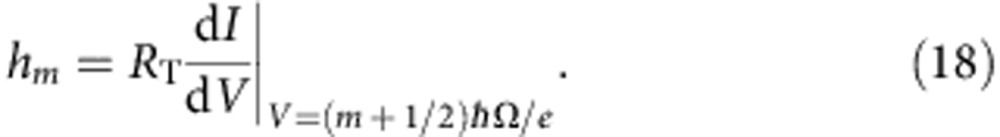


The height of these plateaus is directly linked to *P*_tot_(*E*). Consider the simplest case where *T*_el_→0 and *κ*→0 (so that the energy of a cavity photon is precisely *ℏ*Ω). By combining equations (1) and (5), the normalized first plateau height (that is, zero-bias conductance) is


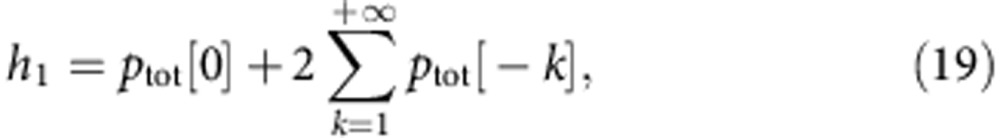


while the height of subsequent plateaus is


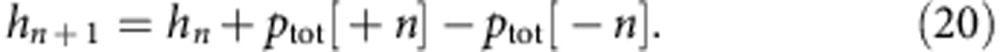


The behaviour of d*I*/d*V* with *V* allows us to easily extract (*p*_tot_[+*n*]−*p*_tot_[−*n*]), the probability difference between an *n*-photon absorption and emission processes.

To extract the sum of these probabilities (and hence reconstruct the full distribution *p*_tot_[*n*]), one also needs to measure the finite-frequency current noise of the junction. We define the (symmetrized) finite-frequency current noise of the junction as


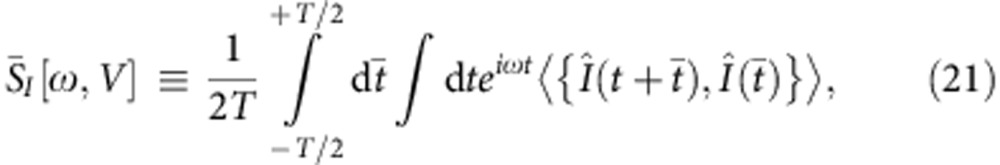


where 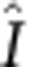 is the junction current operator. The average over 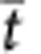 is to pick out the stationary part of the noise (with the averaging time 
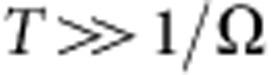
). This noise spectral density depends both on the drain-source voltage *V* and the cavity state. In the tunnelling regime, and for *eV*<*ℏ*Ω, one finds that the excess noise 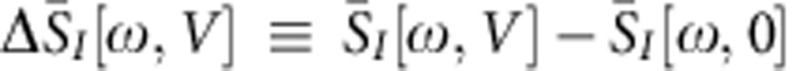 exhibits regular peaks as a function of *ω*, occurring at *ω*=*m*Ω (ref. 42). These noise peaks again correspond to the turning on and off of photon-assisted transport processes. In the low temperature, low-dissipation case, the heights of these peaks can be directly related to *p*_tot_[*n*] (ref. 42)


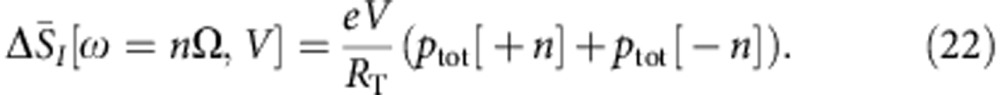


Thus, measuring both the steps in the differential conductance and the peaks in the frequency-dependent excess current noise allow one to directly extract the probabilities *p*_tot_[*n*]. As mentioned in the main text (c.f. equation (14)), once one has measured *p*_tot_[*m*] (as described above), one can explicitly extract the values of quasi-probabilities *p*_occ_[*m*].

### Detecting negative quasi-probability

The distribution *p*_tot_[*k*] governing photon absorption/emission events is a convolution of *p*_0_[*k*] (absorption because of vacuum noise) and *p*_occ_[*k*] (absorption and emission because of the presence of photons in the cavity). *p*_0_[*k*] is a Poisson distribution and is completely determined by the cavity frequency and dimensionless impedance *ρ*; *ρ* could be extracted by, for example, measuring d*I*/d*V* for a ground-state cavity. This then gives a route for detecting the negative values of *p*_occ_[*k*] associated with quantum states. By using the known behaviour of *p*_0_[*k*], one can derive general bounds on the differential conductance and excess noise that must be satisfied *for any* positive definite *p*_occ_[*k*]. A violation of such a bound provides direct evidence of negativity in *p*_occ_[*k*], and hence of the nonclassical nature of the cavity state.

For example, for values of 
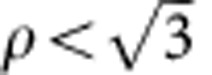
, one can derive a minimum possible value for *h*_2_ consistent with a positive *P*_occ_(*E*) (see Supplementary Note 3):


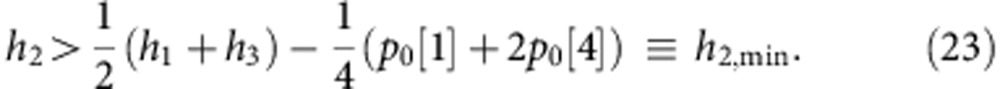


Heuristically, this bound tells us that for positive weights *p*_occ_[*k*], the second conductance step cannot be arbitrarily lower than the average height of the first and third steps. As shown in Fig. 3, this inequality is violated if one prepares a *ρ*=0.5 cavity in a *n*=2 Fock state. Thus, the differential conductance of the junction gives a direct signature of nonclassical behaviour.

In a similar manner, one can derive bounds on the behaviour of *p*_tot_[*k*] that are true for any positive definite *p*_occ_[*k*]; such bounds are in general even more easily violated by the presence of negativity in *p*_occ_[*k*]. For example, for *ρ*<2, one finds that any positive definite *p*_occ_[*k*] must yield (see Supplementary Note 3):


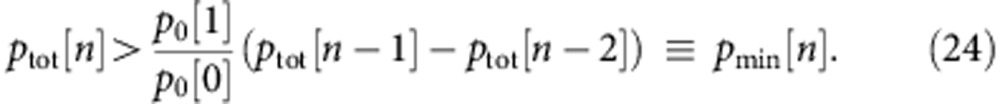


For *n*=1, this bound is violated for a cavity with *ρ*=1.4 prepared in a *r*=1 squeezed vacuum state (see Fig. 5a). This violation can be detected experimentally, as *p*_tot_[−1] can be extracted from the behaviour of d*I*/d*V* and Δ*S*_*I*_[*ω*,*V*], c.f. equations (20) and (22).

### Heisenberg–Langevin equation

A damped, driven cavity can be described using standard input–output theory^30^, with the cavity equation of motion


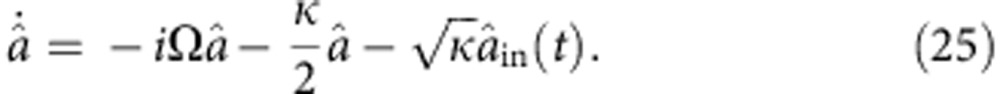


Here 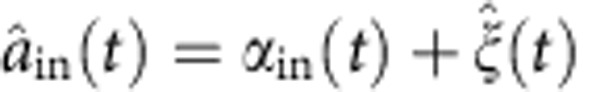 describes the input field on the cavity: it has an average part *α*_in_(*t*) that describes the classical amplitude of the drive, and a noise part 
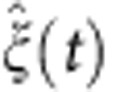
 that describes both thermal and quantum noise incident on the cavity. As with standard input–output theory treatments, this noise is taken to be operator-valued Gaussian white noise. For a coherent state drive, 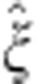 describes vacuum noise, and the average cavity amplitude is 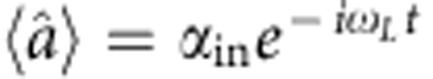. Squeezed input noise can be simply included in the formalism; it corresponds to anomalous correlators 
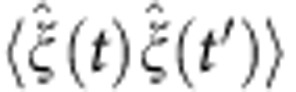
 being non-zero.

As the input noise is Gaussian and the cavity has no nonlinearities, the cavity will also be in a Gaussian state. As a result, the phase–phase correlator in equation (3) is completely determined by two-point correlation functions and is thus easily found from the solution of equation (25).

### Effects of finite temperature

**Figure 6:**
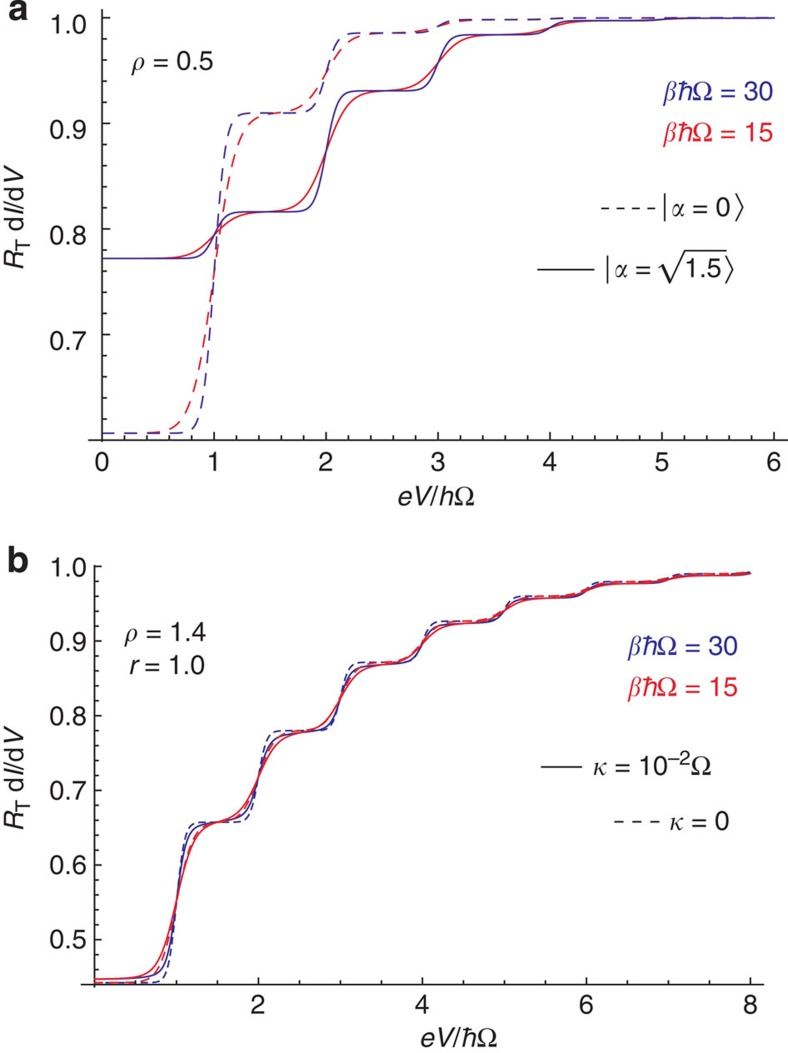
Temperature effects. (**a**) Effects of the temperature on the differential conductance of a tunnel junction coupled to a ground-state closed cavity (dashed line) and to a closed cavity in a coherent state of amplitude 
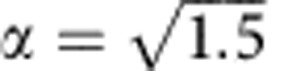
. Parameters are as indicated in the figure, and *β*=1/*k*_B_*T*, where *T* is the temperature of both the cavity and electronic leads. The heights of the plateaus are not affected by the temperature; however, the smearing of transitions decreases their effective width. (**b**) Same as **a**, but for a cavity prepared in a squeezed state with (solid line) and without damping (dashed line). For these parameters, the smearing of transitions between conductance plateaus is more affected by the non-zero cavity damping *κ* than by temperature.

Our discussion in the main text focused on the case where the electronic leads of the tunnel junction are at zero temperature. We stress that equations (1) and (6) remain valid even if the electronic temperature is non-zero. Qualitatively speaking, the effect of finite temperature on the lead electrons is to smear the sharp Fermi step in their energy distribution function; the steps now have a finite slope proportional to (*k*_B_*T*)^−1^. This smearing of the Fermi step directly leads to a broadening of the sharp steps seen in the differential conductance versus dc voltage *V* (see, for example, Fig. 6). It thus follows that to resolve clear plateaus in the differential conductance, one requires 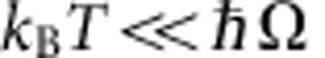. This condition is also consistent with our desire to have the cavity prepared in an interesting quantum state, which requires that the number of thermal quanta in the resonator 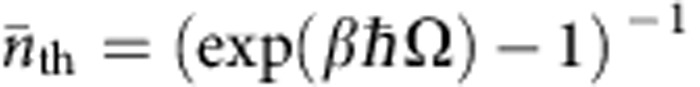 be extremely small.

For concreteness, we consider the realistic case of a microwave cavity with frequency Ω/2*π* in the range 5–10 GHz and an environment temperature in the range 15–30 mK. For these parameters, *βℏ*Ωε[15,30], implying that the thermal number of quanta in the cavity is exponentially suppressed. The main influence of temperature will thus be through the electrons and the smearing of the lead Fermi functions, which manifests itself in the temperature dependence of the tunnelling rates Γ(*E*). The net result is that the height of the various plateaus in the differential conductance are not affected by temperature; however, the transitions between them are rounded off. This behaviour is shown explicitly in Fig. 6.

It is also interesting to consider the effect of having a thermal population of photons in the cavity. The simplest situation is where a cavity is driven by thermal noise via an input port; in this case *P*_occ_(*E*) reduces to the convolution of two Poisson distribution, one for absorption and the other one for emission (see equation (15)). Setting *ℏ*=1, we have


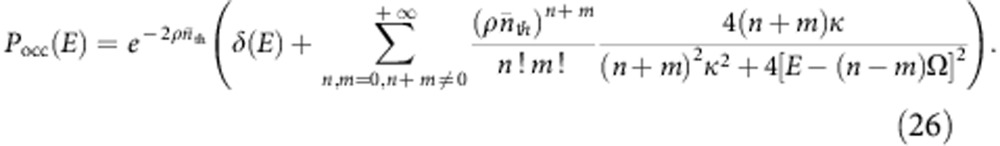


In the limit *κ*→0, this reduces to the well-known DCB expression for a resonator in thermal equilibrium^20^.

Another interesting case is where the damped cavity is driven both by a coherent tone and by thermal noise. In this case the cavity will be in a displaced thermal state, and *P*_occ_[*E*] reduces to the convolution of the thermal distribution given above with the Tien–Gordon distribution (see equation (11)). Again, setting *ℏ*=1, we find


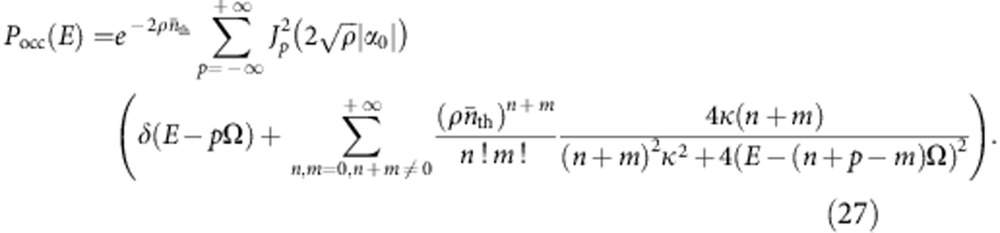


Finally, consider the case of a squeezed state. In the main text, we consider the ideal case where the cavity is driven by a pure squeezed input field. This calculation is easily generalized to the case where the input field describes a squeezed thermal state with thermal population 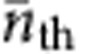. For the small levels of squeezing considered here (that is, *r*=1 in Fig. 5), even thermal populations 
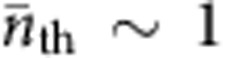
 have a negligible influence. This behaviour is shown in Fig. 6.

### Transport-induced cavity dissipation

The discussion in the main text focused on regimes where the conductor makes a minimal backaction disturbance to the state of the cavity; as discussed, such backaction effects formally play no role in determining the junction current to lowest order in the tunnelling amplitude. A full treatment of backaction effects (that is, working beyond the tunnelling approximation while still allowing for 
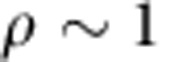
) is beyond the scope of this article and will be the focus of future work. Nonetheless, one can use our approach to estimate the size of such backaction effects in the weak-tunnelling regime; we do this here. The results we obtain are consistent with previous studies of related backaction effects in nanoelectromechanical systems where a tunnel junction is *weakly* coupled to a mechanical resonator^43^,^44^,^45^; such weak-coupling theory would rigorously only apply to 
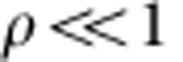
 in our system.

In general, the tunnel junction will act as an additional dissipative reservoir coupled to the cavity; for weak coupling and a high-*Q* cavity, it is equivalent to a Gaussian bath held at some effective temperature (see, for example, refs 43, 44). One effect will thus be an increase in the cavity dissipation rate from *κ* to *κ*+*κ*′. If we simply modelled the tunnel junction as an Ohmic resistance (modelled as an infinite oscillator bath, as per the Caldeira–Leggett approach^46^), we would find *κ*′=1/*R*_T_*C*. The approaches of refs 43, 44 yield an identical expression. This dissipation can be expressed as *κ*′=*ρ*Ω*R*_K_/*R*_T_*π*. We thus see that *κ*′ is negligible compared with *κ* as long as *πR*_T_/*ρR*_K_ is larger than the quality factor of the cavity.

One must also consider the heating of the cavity because of the tunnel junction, that is, what is the effect of the fluctuations associated with the dissipation rate *κ*′? To estimate this heating, consider first an undamped cavity and suppose we have calculated *p*_tot_[*k*] for some given cavity state. We now want to understand how photon-assisted transitions involving the junction lead to heating (or cooling) of the cavity. Using a Golden rule approach, the rate of change of the average cavity photon number because of such transitions will be given by:


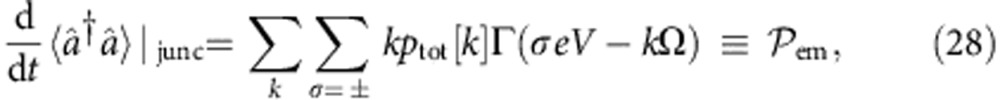


where 
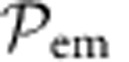
 denotes the power emitted by the junction to the cavity (in units of cavity quanta per unit time). Consider the case *T*_el_→0 and *eV*<*ℏ*Ω. In this case, one finds easily:


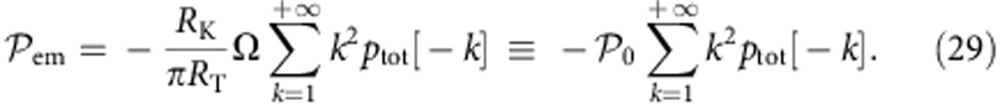


We see that in this low-voltage regime, there is a net power transfer from the cavity to the conductor; its value involves a weighted sum over all processes where a photon is absorbed from the cavity. This is consistent with the results of refs 43, 44, which show that for weak couplings and for low voltages, the resonator sees the conductor as an effective thermal reservoir with an effective temperature much less than *ℏ*Ω/*k*_B_. For 
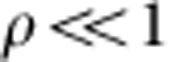
, the sum in equation (29) necessarily scales as *ρ* (as such processes are impossible without having a coupling between the cavity and conductor). In contrast, for the regime of interest here 
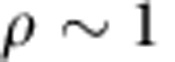
, and the value of the summation could be of order 1 depending on the cavity state. The scale for 
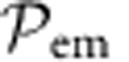
 is thus set by the rate 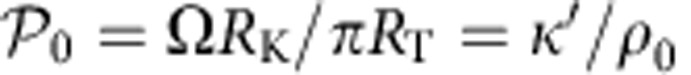; as expected, the weaker the tunnelling (that is, the larger the tunnel resistance *R*_T_), the smaller this rate becomes.

One can, in an analogous manner, calculate 
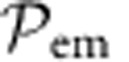
 for larger junction voltages satisfying *eV*>*ℏ*Ω. We are interested in values of 
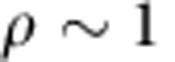
 and in voltages that are at most only a few times larger than *ℏ*Ω. In this regime (and for the kinds of cavity states considered in the main text), one again finds that the scale of 
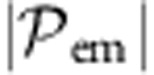
 is set by 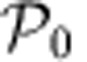. This is shown explicitly in the Methods and in Supplementary Fig. 1 and where we explicitly calculate 
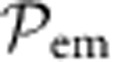
 for a variety of states (see Supplementary Figs 1 and 2).

Having understood the power flow into and out of the cavity, we can now calculate the resulting change in the average cavity photon number, Δ*n*_cav_, using a simple rate equation. We find that


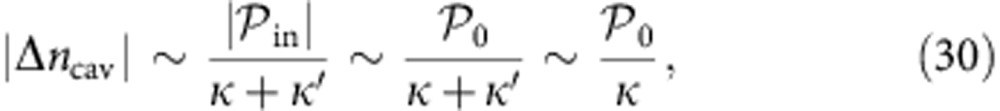


where we have assumed that the intrinsic cavity dissipation *κ* dominates any additional damping *κ*′ because of the coupling to the junction. Insisting that Δ*n*_cav_ is smaller than a single quantum thus results in the condition:


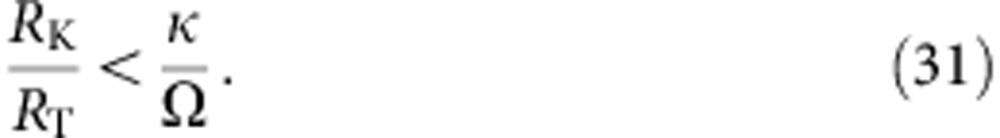


Thus, if we use a cavity with *κ*=10^−2^Ω, the above estimate tells us that the junction resistance needs to be much larger than ~10^2^*R*_K_.

## Author contributions

All authors participated in the conception and planning of the project and were involved in the analysis and interpretation of the results. More specifically, J.G. identified experimentally relevant parameter regimes and set-ups; J.-R.S., M.J.W., P.S. and A.A.C. were involved in the derivation of the theoretical results. A.A.C., J.-R.S., M.J.W. and P.S. wrote the manuscript. A.A.C. and P.S. supervised the project.

## Additional information

**How to cite this article:** Souquet, J.-R. *et al*. Photon-assisted tunnelling with nonclassical light. *Nat. Commun.* 5:5562 doi: 10.1038/ncomms6562 (2014).

## Supplementary Material

Supplementary Information Supplementary Figures 1-2, Supplementary Notes 1-3 and Supplementary References.

## References

[b1] Tien P. K. & Gordon J. P. Multiphoton process observed in the interaction of microwave fields with the tunneling between superconductor films. Phys. Rev. 129, 647 (1963).

[b2] Tucker J. R. & Feldman M. J. Quantum detection at millimeter wavelengths. Rev. Mod. Phys. 57, 1055 (1985).

[b3] Kouwenhoven L. P. . Observation of photon-assisted tunneling through a quantum dot. Phys. Rev. Lett. 73, 3443 (1994). 10057382 10.1103/PhysRevLett.73.3443

[b4] Gabelli J. & Reulet B. Dynamics of quantum noise in a tunnel junction under ac excitation. Phys. Rev. Lett. 100, 026601 (2008). 18232900 10.1103/PhysRevLett.100.026601

[b5] Hoist T., Esteve D., Urbina C. & Devoret M. H. Effect of a transmission line resonator on a small capacitance tunnel junction. Phys. Rev. Lett. 73, 3455 (1994). 10057385 10.1103/PhysRevLett.73.3455

[b6] Basset J., Bouchiat H. & Deblock R. Emission and absorption quantum noise measurement with an on-chip resonant circuit. Phys. Rev. Lett. 105, 166801 (2010). 21230992 10.1103/PhysRevLett.105.166801

[b7] Hofheinz M. . Bright side of the coulomb blockade. Phys. Rev. Lett. 106, 217005 (2011). 21699333 10.1103/PhysRevLett.106.217005

[b8] Prey T. . Dipole coupling of a double quantum dot to a microwave resonator. Phys. Rev. Lett. 108, 046807 (2012). 22400878 10.1103/PhysRevLett.108.046807

[b9] Petersson K. D. . Circuit quantum electrodynamics with a spin qubit. Nature 490, 380–383 (2012). 23075988 10.1038/nature11559

[b10] Delbecq M. . Coupling a quantum dot, fermionic leads, and a microwave cavity on a chip. Phys. Rev. Lett. 107, 256804 (2011). 22243102 10.1103/PhysRevLett.107.256804

[b11] Gasse G., Lupien C. & Reulet B. Observation of squeezing in the electron quantum shot noise of a tunnel junction. Phys. Rev. Lett 111, 136601 (2013). 24116798 10.1103/PhysRevLett.111.136601

[b12] Jin P.-Q., Marthaler M., Cole J. H., Shnirman A. & Schon G. Lasing and transport in a quantum-dot resonator circuit. Phys. Rev. B 84, 035322 (2011).

[b13] Bergenfeldt C. & Samuelsson P. Microwave quantum optics and electron transport through a metallic dot strongly coupled to a transmission line cavity. Phys. Rev. B 85, 045446 (2012).

[b14] Bergenfeldt C. & Samuelsson P. Nonlocal transport properties of nanoscale conductor-microwave cavity systems. Phys. Rev. B 87, 195427 (2013).

[b15] Cottet A., Mora C. & Kontos T. Mesoscopic admittance of a double quantum dot. Phys. Rev. B 83, 121311 (2011).

[b16] Odintsov A. A. Effect of dissipation on the characteristics of small-area tunnel junctions: application of the polaron model. Sov. Phys. JETP 67, 1265 (1988).

[b17] Nazarov Y. V. Anomalous current-voltage characteristics of tunnel junctions. Sov. Phys. JETP 68, 561 (1989).

[b18] Devoret M. H. . Effect of the electromagnetic environment on the Coulomb blockade in ultrasmall tunnel junctions. Phys. Rev. Lett. 64, 1824 (1990). 10041498 10.1103/PhysRevLett.64.1824

[b19] Girvin S. M., Glazman L. I., Johnson M., Penn D. R. & Stiles M. D. Quantum fluctuations and the single-junction Coulomb blockade. Phys. Rev. Lett. 64, 3183 (1990). 10041919 10.1103/PhysRevLett.64.3183

[b20] Ingold G.-L. & Nazarov Y. Single Charge Tunneling Vol. 7, eds Grabert H., Devoret M. H. 935Plenum (1992).

[b21] Delsing P., Likharev K. K., Kuzmin L. S. & Claeson T. Effect of high-frequency electrodynamic environment on the single-electron tunneling in ultrasmall junctions. Phys. Rev. Lett. 63, 1180 (1989). 10040490 10.1103/PhysRevLett.63.1180

[b22] Geerligs L. J., Anderegg V. F., Jeugd C. A. v. d., Romijn J. & Mooij J. E. Influence of dissipation on the coulomb blockade in small tunnel junctions. Europhys. Lett. 10, 79 (1989).

[b23] Cleland A. N., Schmidt J. M. & Clarke J. Charge fluctuations in small-capacitance junctions. Phys. Rev. Lett. 64, 1565 (1990). 10041430 10.1103/PhysRevLett.64.1565

[b24] Altimiras C., Gennser U., Cavanna A., Mailly D. & Pierre F. Experimental test of the dynamical Coulomb blockade theory for short coherent conductors. Phys. Rev. Lett. 99, 256805 (2007). 18233546 10.1103/PhysRevLett.99.256805

[b25] Parmentier F. . Strong back-action of a linear circuit on a single electronic quantum channel. Nat. Phys. 7, 935–938 (2011).

[b26] Armour A., Blencowe M., Brahimi E. & Rimberg A. Universal quantum fluctuations of a cavity mode driven by a Josephson junction. Phys. Rev. Lett. 111, 247001 (2013). 24483692 10.1103/PhysRevLett.111.247001

[b27] Li S.-X. & Kycia J. B. Applying a direct current bias to superconducting microwave resonators by using superconducting quarter wavelength band stop filters. Appl. Phys. Lett. 102, 242601 (2013).

[b28] Chen F., Sirois A. J., Simmonds R. W. & Rimberg A. J. Introduction of a dc bias into a high-Q superconducting microwave cavity. Appl. Phys. Lett. 98, 132509 (2011).

[b29] Nazarov Y. & Blanter Y. Quantum Transport: Introduction to Nanoscience Cambridge (2009).

[b30] Clerk A. A., Devoret M. H., Girvin S. M., Marquardt F. & Schoelkopf R. J. Introduction to quantum noise, measurement, and amplification. Rev. Mod. Phys. 82, 1155 (2010).

[b31] Devoret M. H., Girvin S. M. & Schoelkopf R. J. Circuit-QED: How strong can the coupling between a Josephson junction atom and a transmission line resonator be? Armalen Phys. 16, 767–779 (2007).

[b32] Masluk N. A., Pop I. M., Kamal A., Minev Z. K. & Devoret M. H. Microwave characterization of josephson junction arrays: implementing a low loss superinductance. Phys. Rev. Lett. 109, 137002 (2012). 23030112 10.1103/PhysRevLett.109.137002

[b33] Altimiras C. . Dynamical Coulomb blockade of shot noise. Phys. Rev. Lett. 112, 236803 (2014). 24972223 10.1103/PhysRevLett.112.236803

[b34] Povatos J., Cirac J. & Zoller P. Quantum reservoir engineering with laser cooled trapped ions. Phys. Rev. Lett. 77, 4728 (1996). 10062616 10.1103/PhysRevLett.77.4728

[b35] Murch K. . Cavity-assisted quantum bath engineering. Phys. Rev. Lett. 109, 183602 (2012). 23215278 10.1103/PhysRevLett.109.183602

[b36] Shankar S. . Autonomously stabilized entanglement between two superconducting quantum bits. Nature 504, 419–422 (2013). 24270808 10.1038/nature12802

[b37] Gerry C. C. & Knight P. L. Introductory Quantum Optics Cambridge (2005).

[b38] Holland E. . inMarch Meeting 2014 Abstract Bulletin of the American Physical Society (2014).

[b39] Didier N., Qassemi F. & Blais A. Perfect squeezing by damping modulation in circuit quantum electrodynamics. Phys. Rev. A 89, 013820 (2014).

[b40] Murch K. W., Weber S. J., Beck K. M., Ginossar E. & Siddiqi I. Reduction of the radiative decay of atomic coherence in squeezed vacuum. Nature 499, 62–65 (2013). 23823794 10.1038/nature12264

[b41] Zakka-Bajjani E. . Experimental test of the high-frequency quantum shot noise theory in a quantum point contact. Phys. Rev. Lett. 99, 236803 (2007). 18233393 10.1103/PhysRevLett.99.236803

[b42] Souquet J.-R., Safi I. & Simon P. Dynamical Coulomb blockade in an interacting ID system coupled to an arbitrary-environment. Phys. Rev. B 88, 205419 (2013).

[b43] Mozyrsky D. & Martin I. Quantum-classical transition induced by electrical measurement. Phys. Rev. Lett. 89, 018301 (2002). 12097073 10.1103/PhysRevLett.89.018301

[b44] Clerk A. A. & Girvin S. M. Shot noise of a tunnel junction displacement detector. Phys. Rev. B 70, 121303 (2004).

[b45] Bennett S. D. & Clerk A. A. Full counting statistics and conditional evolution in a nanoelectromechanical svstem. Phys. Rev. B 78, 165328 (2008).

[b46] Caldeira A. O. & Leggett A. J. Quantum tunnelling in a dissipative system. Ann. Phys. 149, 374–456 (1983).

