# Supplementary material for: Photon-assisted tunnelling with nonclassical light
Source: Nat Commun. 2014 Nov 26;5:5562. doi: 10.1038/ncomms6562 (PMC4263132; doi:10.1038/ncomms6562)
Supplement: Supplementary Information — Supplementary Figures 1-2, Supplementary Notes 1-3 and Supplementary References. [file ncomms6562-s1.pdf]

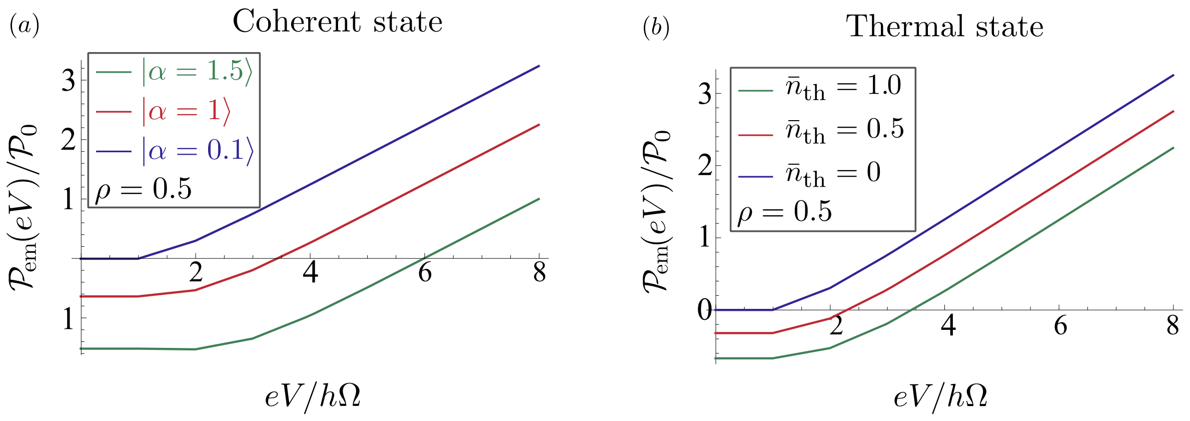

Supplementary Figure 1. (a) Power emitted by the junction $\mathcal{P}_{\rm em}$ to the cavity in units of $\mathcal{P}_0$ (c.f. Equation (29)), as a function of the dc junction bias voltage V. Each curve corresponds to the cavity being in a coherent state with a given amplitude α. For small V, the junction acts like a low-temperature bath and acts to cool the cavity (it absorbs energy). For higher biases, the effective temperature of the junction increases, and there is a net energy flow from the junction to the cavity. For the range of voltage considered, the emitted power is of order of $\mathcal{P}_0$. All curves correspond to zero temperature and zero cavity damping. (b) Same quantity, now for a thermal state in the cavity, for different choices of the thermal photon number $\bar{n}_{\rm th}$.

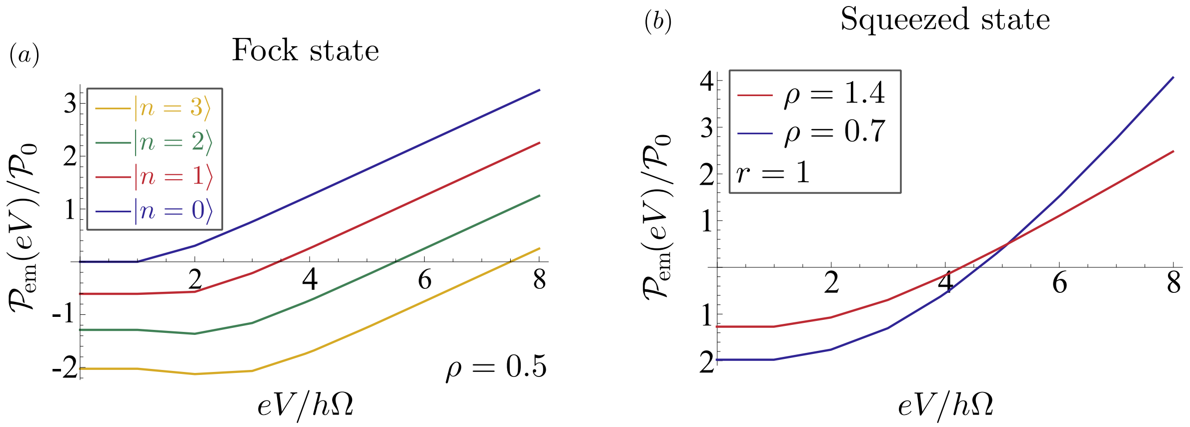

Supplementary Figure 2. Similar to Supplementary Figure 1, but now we take the cavity to be in a non-classical state (as specified in the legend); all curves are for zero temperature and for zero cavity damping.

Supplementary Note 1: $P(E)$ THEORY FOR A GENERAL NON-EQUILIBRIUM ENVIRONMENT.

General derivation

In this section, we present the derivation of Equation (1), which generalises the standard $P(E)$ theory for tunnelling in the presence of an electromagnetic environment to cases where the environment is in an arbitrary time-dependent state. We consider a tunnel junction between left and right metallic reservoirs which is voltage biased both by a fixed dc voltage V and by a voltage created by a bosonic electromagnetic environment. The Hamiltonian is

$$\hat{H} = \hat{H}_{\rm el} + \hat{H}_{\rm env} + \hat{H}_{\rm tun}, \tag{1}$$

where $\hat{H}_{\rm el}$ and $\hat{H}_{\rm env}$ denote the Hamiltonians of the leads and of the cavity respectively, and $\hat{H}_{\rm tun}$ the tunnelling between leads. Making the usual gauge transformation to include voltages directly in $\hat{H}_{\rm tun}$, we have ($\hbar = 1$ here and

throughout the Supplementary Information)

$$\hat{H}_{\rm tun} = w\sum_{k,q}\hat{c}^{\dagger}_{R,k}\hat{c}_{L,q}e^{ieVt}e^{i\hat{\varphi}(t)} + h.c. \equiv \hat{W}e^{ieVt}e^{i\hat{\varphi}(t)} + h.c., \tag{2}$$

where w is the tunnel matrix element, $\hat{c}_{\alpha,k}$ is the destruction operator for a single particle state k in lead α, and $\hat{\varphi}(t)$ is the phase operator associated with the environment voltage (see Equation (4)). The tunnel resistance $R_{\rm T}$ of the junction is given by $1/R_{\rm T} = 2\pi(e^2/h)w^2\rho_0^2$, where ρ_0 is the lead density of states at the Fermi energy. As usual, the current operator is given by $\hat{I} = -i\left(\hat{W} - \hat{W}^{\dagger}\right)$. Using standard quantum linear response theory (i.e. the Kubo formula), the current at time t to order w^2 is given by

$$\langle\hat{I}(t)\rangle = e\left[\Gamma_+(t) - \Gamma_-(t)\right], \tag{3}$$

$$\Gamma_\sigma(t) = {\rm Re}\int_{-\infty}^{t-t_0} d\tau\, G_{\rm el}(\tau)G_{\rm env}(t,\tau;\sigma)e^{i\sigma eV\tau}, \tag{4}$$

where $\sigma = \pm$ corresponds to electron tunnelling respectively from left to right and right to left, and where the relevant electronic and environment Green functions are evaluated in the absence of tunnelling, and are given by

$$G_{\rm el}(\tau) = -i\langle\hat{W}(\tau)\hat{W}^{\dagger}(0)\rangle, \tag{5}$$

$$G_{\rm env}(t,\tau;\sigma) = -i\theta(\tau)\langle e^{i\sigma\hat{\varphi}(t)}e^{-i\sigma\hat{\varphi}(t-\tau)}\rangle, \tag{6}$$

We have used the fact that the uncoupled electronic system is time translationally invariant (while we have not assumed this about the bosonic environment). Here, t_0 corresponds to the time at which the tunnel Hamiltonian was switched on; we let $t_0 \to -\infty$.

Next, note that for free electrons:

$$G_{\rm el}[\omega] = -i\Gamma[\omega] = -i\frac{1}{e^2R_{\rm T}}\frac{\omega}{1-\exp(-\omega/k_{\rm B}T_{\rm el})}, \tag{7}$$

Using the convolution theorem to evaluate Supplementary Equation (4), we recover Equation (1).

Time-independent environment

For the standard case where the environment is in a time-independent state, environmental correlation functions are time-translation invariant, and hence $G_{\rm env}(t,\tau;\sigma)$ (c.f. Equation (2)) becomes independent of the time t. It is then easy to show that Equation (3) reduces to

$$P_{\rm tot}(E;\sigma) = \int_{-\infty}^{+\infty} d\tau e^{iE\tau}\langle e^{i\sigma\hat{\varphi}(\tau)}e^{-i\sigma\hat{\varphi}(0)}\rangle. \tag{8}$$

Further, $P(E)$ theory is usually applied to situations where the environment is invariant under $\hat{\varphi} \to -\hat{\varphi}$. In this case, there is no dependence on σ, and one recovers the standard formula for the $P(E)$ function as the Fourier transform of the environmental phase-phase correlator.

Positivity of time-averaged $P_{\rm tot}(E;t)$: closed cavity

For a general time-dependent environment, the function $P_{\rm tot}(E;t,\sigma)$ will be explicitly time-dependent and could take on negative values. We now focus on the simple case where the environment is a closed cavity, prepared in some arbitrary state. The cavity Hamiltonian is time independent, and all dependence on t arises from preparing the system in a non-stationary state (i.e. the cavity density matrix $\hat{\rho}_{\rm cav}$ is not diagonal in the basis of energy eigenstates). In this case, we write $P_{\rm tot}(E;t,\sigma)$, using the convolution theorem, in the following form :

$$P_{\rm tot}(\omega;t,\sigma) = \frac{-{\rm Im}}{\pi}\int d\omega'\left(-i\pi\delta(\omega-\omega') + \frac{1}{\omega-\omega'}\right)\Lambda(\omega',t;\sigma), \tag{9}$$

$$\Lambda(\omega;t,\sigma) = \int_{-\infty}^{+\infty} d\tau\langle e^{i\sigma\hat{\varphi}(t)}e^{-i\sigma\hat{\varphi}(t-\tau)}\rangle e^{i\omega\tau} \tag{10}$$

$$= \sum_{i,i',f=0}^{+\infty}\langle i|e^{i\sigma\hat{\varphi}}|f\rangle\langle f|e^{-i\sigma\hat{\varphi}}|i'\rangle\langle i'|\hat{\rho}|i\rangle\delta(E_f - E_{i'} - \omega)e^{-i(E_{i'}-E_i)t}. \tag{11}$$

Here, $|j\rangle$ labels energy eigenstates of the system with corresponding eigenvalues $E_j = \hbar\Omega(j+1/2)$.

In general, $P_{\rm tot}(\omega;t,\sigma)$ and $\langle \hat{I}(t)\rangle$ will oscillate as a function of t with a period $2\pi/\Omega$. To obtain the dc current, we will average t over one period:

$$P_{\rm tot}(E) \equiv \frac{1}{t_{\rm avg}} \int_{-t_{\rm avg}/2}^{+t_{\rm avg/2}} dt' P_{\rm tot}(E, t+t';\sigma), \tag{12}$$

where $t_{\rm avg} = 2\pi/\Omega$. The time average kills all terms in Supplementary Equation (11) except those where $E_i = E_{i'}$. As the cavity has no degeneracies in its spectrum, it immediately follows that the only terms in $\Lambda(\omega,t;\sigma)$ surviving the time average have $i = i'$, and are thus proportional to matrix elements of the form $|\langle f|e^{-i\sigma\hat{\varphi}}|i\rangle|^2$. It thus follows that all contributing terms to Λ are positive definite, and thus so is $P_{\rm tot}(E)$. Further, such matrix elements are independent of whether $\sigma = \pm 1$; hence, $P_{\rm tot}$ is independent of σ.

Positivity of time-averaged $P_{\rm tot}(E;t)$: general case

In the general case, where the total $P(E)$ function is not periodic, we define the time-averaged $P(E)$ function as:

$$\bar{P}_{\rm tot}(E;\sigma) \equiv \lim_{T\to\infty} \frac{1}{T} \int_{-T/2}^{+T/2} dt\, P_{\rm tot}(E;t,\sigma), \tag{13}$$

where $P_{\rm tot}(E;t,\sigma)$ is defined in Equation (3). From Supplementary Equation (9), we see that $\bar{P}_{\rm tot}(E;\sigma)$ is necessarily positive definite if the quantity

$$\bar{\Lambda}(\omega,\sigma) = \lim_{T\to\infty} \frac{1}{T} \int_{-T/2}^{T/2} dt \Lambda(\omega;t,\sigma), \tag{14}$$

is positive definite, where $\Lambda(\omega;t,\sigma)$ is defined in Supplementary Equation (10) above. To show this, we first define

$$\hat{A}[\omega] \equiv \frac{1}{\sqrt{T}} \int_{-T/2}^{T/2} dt'\, \hat{A}(t') e^{i\omega t'} \tag{15}$$

for some arbitrary Heisenberg-picture operator $\hat{A}(t)$. It immediately follows that:

$$Q(\omega,T) \equiv \left\langle \hat{A}[\omega] \left(\hat{A}[\omega]\right)^\dagger \right\rangle \geq 0. \tag{16}$$

We can express $Q(\omega,T)$ as

$$Q(\omega,T) = \frac{1}{T} \int_{-T/2}^{T/2} dt \int_{t-T/2}^{t+T/2} d\tau \langle \hat{A}(t) \hat{A}^\dagger(t-\tau)\rangle e^{i\omega\tau}. \tag{17}$$

Assuming that the correlation function in Supplementary Equation (17) has a finite correlation time (i.e. it decays for sufficiently large $|\tau|$), when taking the limit $T\to\infty$ we can safely replace the bounds of the τ integration by $\pm\infty$. Making the choice $\hat{A}(t) = e^{i\sigma\hat{\varphi}(t)}$, we then have

$$\bar{\Lambda}(\omega,\sigma) \equiv \lim_{T\to\infty} Q(\omega,T) \quad \Longrightarrow \bar{\Lambda}(\omega,\sigma) \geq 0. \tag{18}$$

This proves that $\bar{P}_{\rm tot}(E;\sigma)$ must be positive definite.

Finally, note that in the cases of interest in the main text, $P_{\rm tot}(E;t,\sigma)$ is a periodic function of t. In this case the infinite-time average over t in Supplementary Equation (13) is equivalent to averaging t over a single period.

Supplementary Note 2: $P_{\rm occ}(E)$ FOR VARIOUS CLOSED-CAVITY STATES.

In what follows, it will be useful to introduce the inverse Fourier transform of $P_{\rm occ}(E)$, $P_{\rm occ}(\tau)$, via

$$P_{\rm occ}(E) = \int_{-\infty}^{+\infty} d\tau e^{iE\tau} P_{\rm occ}(\tau). \tag{19}$$

For a closed cavity, the kernel $P_{\rm occ}(\tau)$ is directly related to the characteristic function χ of the Glauber-Sudarshan P function $\mathcal{K}(\alpha)$, c.f. Equations (16)-(17). Recall that $\mathcal{K}(\alpha)$ allows one to represent a given cavity state in phase space, c.f. Equation (8).

Thermal state

Consider a closed cavity in a thermal state having an average photon occupancy $\bar{n}_{\rm th} = (e^{\Omega/k_{\rm B}T} - 1)^{-1}$. Using Equations (16),(17), one finds

$$P_{\rm occ}(\tau) = \exp\left(2\rho\bar{n}_{\rm th}(\cos\Omega\tau \; - 1)\right). \tag{20}$$

The quasiprobability distribution $P_{\rm occ}(E)$ is then

$$P_{\rm occ}(E) = e^{-2\rho\bar{n}_{\rm th}} \sum_{n,m=0}^{+\infty} \frac{(\rho\bar{n}_{\rm th})^{n+m}}{n!\,m!} \delta\left[E - (n-m)\hbar\Omega\right] \equiv \sum_n p_{\rm occ,th}[n, \bar{n}_{\rm th}]\delta[E - n\hbar\Omega]. \tag{21}$$

$P_{\rm occ}(E)$ is just the convolution of two Poisson distributions: the first describes the Poisson absorption of photons at rate $\rho\bar{n}_{th}$, the second the Poissonian emission of photons at $\rho\bar{n}_{\rm th}$. It thus has the form of a Skellam distribution. Convolving in the vacuum distribution $P_0(E)$ given in Equation (7), one obtains the final distribution $P_{\rm tot}(E)$. This continues to have the form of a Skellam distribution, and recovers the expression for a thermal cavity which is well known from the standard theory of DCB [1].

Squeezed states

Consider first the case where a cavity is prepared in a pure squeezed state which evolves without dissipation. The initial cavity state is parametrised as:

$$|r,\theta\rangle = \exp\left(re^{i\theta}\hat{a}^\dagger\hat{a}^\dagger - h.c.\right)|0\rangle, \tag{22}$$

where r is the squeeze parameter and the angle θ determines the orientation of the squeezed cavity quadrature. In the absence of dissipation, the $P_{\rm occ}(E)$ function describing a cavity squeezed state can easily be calculated from characteristic function of this state. Before averaging over the observation time t, we have:

$$P_{\rm occ}(\tau,t) = \exp\left[-4\rho\sinh^2 r\sin^2\tfrac{\Omega\tau}{2}\right]\exp\left[2\rho\sinh 2r\;\sin^2\frac{\Omega\tau}{2}\;\cos(2\Omega t+\theta)\right]. \tag{23}$$

$P_{\rm occ}(\tau)$ is obtained by averaging over t. Without loss of generality, we shift the zero of time to absorb the phase θ. It is useful to first Fourier transform in the relative time variable τ, but keep the dependence on observation time t. Note that each factor above (for fixed t) has the same functional dependence on τ as the $P_{\rm occ}(\tau)$ for a thermal state (c.f. Supplementary Equation (20)). One thus obtains a simple convolution of two thermal distributions:

$$P_{\rm occ}(E,t) = \sum_n p_{\rm occ}[n,t]\delta(E - n\hbar\Omega), \tag{24}$$

$$p_{\rm occ}[n,t] = \sum_m p_{\rm occ,th}[n-m, \bar{n}_{\rm th} = \sinh^2 r]\cdot p_{\rm occ,th}[m, \bar{n}_{\rm th} = -\frac{1}{2}\sinh 2r\;\cos 2\Omega t], \tag{25}$$

where the weights $p_{\rm occ,th}[m,\bar{n}_{\rm th}]$ for a thermal distribution are defined in Supplementary Equation (21). Note that the second thermal distribution in the convolution has an effective temperature which is time dependent and *which can be negative* (i.e. for times where $\cos 2\Omega t > 0$). This leads to negativity in $p_{\rm occ}[n,t]$, negativity which can persist even after averaging over the observation time t. It thus is the origin of negativity in $P_{\rm occ}(E)$ for a squeezed state. Further, note that when we average over t, even and odd photon number processes generated by the second thermal distribution will be impacted differently (as for small ρ, $p_{\rm occ,th}[m,\bar{n}_{\rm th}] \propto (\bar{n}_{\rm th})^m$). Thus, the above form also suggests the origin of the even-odd asymmetry in $P_{\rm occ}(E)$ for a cavity squeezed state.

If we time average $P_{\rm occ}(\tau,t)$, we find:

$$P_{\rm occ}(\tau) = e^{-4\rho\sinh^2 r\;\sin^2\frac{\Omega\tau}{2}} I_0\left[2\rho\sinh 2r\;\sin^2\frac{\Omega\tau}{2}\right]. \tag{26}$$

Note that for a highly squeezed state, it is tempting to take the $r\to\infty$ limit, and make the approximations $\sinh^2 r \sim e^{2r}/4$, $\sinh 2r \sim e^{2r}/2$. In this case, we could introduce $\rho_{\rm eff} = \rho e^{2r}/2$, and write:

$$P_{\rm occ}(\tau) \simeq e^{-\rho_{\rm eff}\sin^2\frac{\Omega\tau}{2}} I_0\left[\rho_{\rm eff}\sin^2\frac{\Omega\tau}{2}\right]. \tag{27}$$

One can confirm that in this limit, $P_{\mathrm{occ}}(E)$ never exhibits any negativity. It follows that even for very large squeeze parameters r, the presence of negativity depends crucially on the magnitude of ρ and requires $\rho \sim 1$.

We can also calculate $P_{\mathrm{occ}}(E)$ for an open (i.e. damped) cavity that is prepared in a squeezed state by continuous driving: squeezed input noise is continuously fed into the cavity. In this case, the cavity state is Gaussian, and $P_{\mathrm{occ}}(E)$ is determined by the two-point phase phase correlator; thus easily calculated using standard Heisenberg-Langevin equations. One finds

$$P_{\mathrm{occ}}(\tau,t) = \exp\left[\rho\left(\Lambda_1 + e^{-\kappa|\tau|/2}\Lambda_2\right)\right], \tag{28}$$

where:

$$\Lambda_1 = -2\sinh^2(r) - \sinh 2r\ \cos\Omega\tau\ \cos 2\Omega t,\quad \Lambda_2 = 2\sinh^2 r\ \cos\Omega\tau\ + \sinh 2r\ \cos 2\Omega t. \tag{29}$$

Note that despite the non-zero dissipation, we have undamped oscillations as a function of τ, corresponding to processes where the photon energy is precisely Ω.

Fock states

We denote the $P_{\mathrm{occ}}(\tau,t)$ function for an n photon Fock state as $P_{\mathrm{occ},n}(\tau,t)$. It is given by:

$$P_{\mathrm{occ},n}(\tau,t) = \langle n|e^{\lambda\hat{a}^\dagger}e^{-\bar{\lambda}\hat{a}}|n\rangle = \sum_{p=0}^{+\infty}\frac{1}{p!^2}\langle n|(-|\lambda|^2\hat{a}^\dagger\hat{a})^p|n\rangle, \tag{30}$$

$$= \sum_{p\in\mathbb{N}}\frac{1}{p!}\binom{n}{p}\left(e^{i\Omega\tau} - 1 + e^{-i\Omega\tau} - 1\right)^p, \tag{31}$$

where we introduced $\lambda(\tau,t) = \sqrt{\rho}(e^{i\Omega(t+\tau)} - e^{i\Omega(t-\tau)})$. Note that the characteristic function does not depend on the observation time t, implying that there will be no average ac current across the tunnel junction. Physically, this is a consequence of the rotational invariance of the Fock state phase space distribution. Let us now discuss the above expression for a few specific choices of n.

1. $n = 1$ *Fock state*

For $n = 1$, Supplementary Equation (31) becomes:

$$P_{\mathrm{occ},1}(\tau) = 1 + \rho\left(e^{i\Omega\tau} + e^{-i\Omega\tau} - 2\right). \tag{32}$$

The probability $P_{\mathrm{occ}}(E)$ is just given by the Fourier transform of $P_{\mathrm{occ}}(\tau)$ and reads

$$P_{\mathrm{occ},1}(E) = (1-2\rho)\delta(E) + \rho\left[\delta(E+\hbar\Omega) + \delta(E-\hbar\Omega)\right]. \tag{33}$$

The total $P(E)$ function including vacuum noise, $P_{\mathrm{tot}}(E)$ is given by a simple convolution with $P_0(E)$. In the time domain, we obtain:

$$P_{\mathrm{tot},1}(\tau) = e^{-\rho}\rho e^{i\Omega\tau} + e^{-\rho}(\rho-1)^2 + \sum_{k\geq 1}\frac{e^{-\rho}\rho^k}{(k+1)!}\left(\rho-(k+1)\right)^2 e^{-ik\Omega\tau} = \sum_{k\in\mathbb{Z}}\frac{e^{-\rho}\rho^k}{(k+1)!}\left(L_1^{(k)}(\rho)\right)^2 e^{-ik\Omega\tau}, \tag{34}$$

where the $L_n^{(k)}(\rho)$ are the generalised Laguerre polynomials. Negative probabilities have disappeared in this expression, as expected.

2. $n = 2$ *Fock state*

For $n = 2$, Supplementary Equation (31) becomes:

$$P_{\mathrm{occ},2}(\tau) = 1 + 2\rho\left(e^{i\Omega\tau} + e^{-i\Omega\tau} - 2\right) + \frac{\rho^2}{2}\left(6 + e^{2i\Omega\tau} + e^{-2i\Omega\tau} - 4\left(e^{i\Omega\tau} + e^{-i\Omega\tau}\right)\right) \tag{35}$$

$$= (1 - 4\rho + 3\rho^2) + 2\rho(1-\rho)\left(e^{i\Omega\tau} + e^{-i\Omega\tau}\right) + \frac{1}{2}\rho^2\left(e^{2i\Omega\tau} + e^{-2i\Omega\tau}\right). \tag{36}$$

Again, negative probabilities for both zero and one photon absorption / emission processes are possible.

Convolving in the vacuum absorption distribution $P_0(E)$ and remaining in the time domain, the full $P(E)$ function is given by:

$$\begin{aligned} P_{\mathrm{tot},2}(\tau) \quad &= \tfrac{\rho^2}{2}e^{2i\Omega\tau} + \left[\tfrac{\rho^3}{2} + 2\rho(1-\rho)\right]e^{i\Omega\tau} + \left[\tfrac{\rho^4}{4} + 2\rho^2(1-\rho) + (1-4\rho+3\rho^2)\right] \\ &+ \left[\tfrac{\rho^5}{2!3!} + 2\rho(1-\rho)\left(\tfrac{\rho^2}{2}+1\right) + (1-4\rho+3\rho^2)\rho\right]e^{-i\Omega\tau} \\ + \textstyle\sum_{k\geq 2}&\left[\tfrac{\rho^2}{2}\left(\tfrac{\rho^{k+2}}{(k+2)!} + \tfrac{\rho^{k-2}}{(k-2)!}\right) + 2\rho(1-\rho)\left(\tfrac{\rho^{k+1}}{(k+1)!} + \tfrac{\rho^{k-1}}{(k-1)!}\right) + (1-4\rho+3\rho^2)\tfrac{\rho^k}{k!}\right]e^{-ki\Omega\tau}. \end{aligned} \tag{37}$$

All these terms factorise nicely, and we obtain the simple closed expression for $P_{\mathrm{occ}}(\tau)$:

$$P_{\mathrm{tot},2}(\tau) = e^{-\rho}\sum_{k\in\mathbb{Z}} \frac{2\rho^k}{(k+2)!}\left[L_2^{(k)}(\rho)\right]^2 e^{-ki\Omega\tau}. \tag{38}$$

3. $n > 2$ Fock states

In order to obtain a closed expression for $P_{\mathrm{occ}}(\tau)$ for an arbitrary Fock state, we first make use of the following property of Laguerre polynomials:

$$L_{n+1}(x) = \frac{1}{n+1}\left((2n+1-x)L_n(x) - nL_{n-1}(x)\right). \tag{39}$$

This allows us to write the following recurrence relation between the $P_{\mathrm{tot,n}}(E)$ function for different Fock states :

$$P_{\mathrm{tot},n+1}(\tau) = \frac{1}{n+1}\left[\left(2(n-\rho)+1+\rho\left(e^{i\Omega\tau}+e^{-i\Omega\tau}\right)\right)P_{\mathrm{tot},n}(\tau) - nP_{\mathrm{tot},n-1}(\tau)\right]. \tag{40}$$

We can expand $P_{\mathrm{tot},n}(\tau)$ in the usual manner in terms of weights for k photon absorption/emission processes, using the fact that the maximum number of photons that can be emitted is n:

$$P_{\mathrm{tot},n}(\tau) = \sum_{k\geq -n} p_{\mathrm{tot},n}[k]e^{-ik\Omega\tau}. \tag{41}$$

Inserting this form into Supplementary Equation (40), we now have the following recurrence relations:

$$p_{\mathrm{tot},n+1}[-n-1] = \frac{\rho}{n+1}p_{\mathrm{tot},n}[-n], \tag{42}$$

$$p_{\mathrm{tot},n+1}[-n] = \frac{1}{n+1}\left(\rho p_{\mathrm{tot},n}[-n+1] + (2(n-\rho)+1)\,p_{\mathrm{tot},n}[-n]\right), \tag{43}$$

$$p_{\mathrm{tot},n+1}[k > -n] = \frac{1}{n+1}\left(\rho(p_{\mathrm{tot},n}[k+1] + p_{\mathrm{tot},n}[k-1]) + (2(n-\rho)+1)p_{\mathrm{tot},n}[k] - np_{\mathrm{tot},n-1}[k]\right). \tag{44}$$

Motivated by the case $n = 1$ and $n = 2$, one finds by inspection that the following compact expression

$$p_{\mathrm{tot},n}[k] = \frac{e^{-\rho}\rho^k n!}{(k+n)!}\left[L_n^{(k)}(\rho)\right]^2, \tag{45}$$

satisfies the above recursion relations. This is Equation (13). As one can explicitly verify that this solution is correct for $n = 1$ and $n = 2$, it is thus necessarily unique.

Supplementary Equation (45) shows explicitly that the total probability (i.e. including the contribution of vacuum fluctuations) to absorb or emit any given number of photons remain positive, regardless of the Fock state; this matches our general result. Nonetheless, it remains possible that the weight $p_{\mathrm{tot},n}[k]$ can be exactly zero for $k > -n$; this requires in general a careful tuning of the parameter ρ. Such a cancellation would be *impossible* if $p_{\mathrm{occ},n}[j]$ were all positive. Hence, it serves as direct proof that we have a quantum state in the cavity.

In particular, consider $p_{\mathrm{tot},n}[0]$, the probability that an electron tunnels without any energy exchange with the cavity. This quantity is directly given by the excess current noise at zero frequency, see Equation (22). From the above expressions, we see that $p_{\mathrm{tot},n}[0]$ can be made zero if ρ is tuned to be a root of the nth Laguerre polynomial. The smallest root of the nth Laguerre polynomial goes as $\sqrt{2}/n$, suggesting that the bigger the photon number of the cavity Fock state, the lower the minimum value of ρ needed to see evidence of negativity.

Supplementary Note 3: USING POSITIVITY OF $P_{\rm occ}(E)$ TO BOUND TRANSPORT MEASUREMENTS.

As discussed in the main text and in the methods section, using the fact that $p_0[k]$ is a known distribution (Poissonian with mean ρ) one can obtain inequalities for both the heights of the differential conductance plateaus and for the weights $p_{\rm tot}[k]$ that *must* be satisfied for any cavity state where the quasi-probability distribution $p_{\rm occ}[k]$ is positive definite. As discussed, this will fail to be true when the cavity is prepared in a truly non-classical state, and hence these bounds may be violated by such states. Three such bounds are used in the main text and two of these are given explicitly in the methods section. Here, we derive them all.

Bound on height of second conductance plateau

We first obtain the bound on the height of the second conductance plateau h_2 given in Equation (23) and used in Figure 3. This bound is useful in inferring the non-classicality of the Fock state via a transport measurement. Using Equation (20), expressing $p_{\rm tot}[k]$ as a convolution [c.f. Equations (5)-(9)], and then using the evenness of $p_{\rm occ}[k]$, we have

$$h_2 - h_1 = p_{\rm tot}[+1] - p_{\rm tot}[-1] = p_0[1]p_{\rm occ}[0] + p_0[2]p_{\rm occ}[1] - \sum_{k=2}^{+\infty} \left(p_0[k-1] - p_0[k+1]\right) p_{\rm occ}[k]. \tag{46}$$

For $k \geq 2$ and $\rho^2 < k(k+1)$ we have $p_0[1] > p_0[k-1] - p_0[k+1] > 0$. Therefore, for any $p_{occ}[k]$ distribution that is positive definite, we have

$$h_2 - h_1 > p_0[1]p_{\rm occ}[0] + p_0[2]p_{\rm occ}[1] - p_0[1]\sum_{k=2}^{+\infty} p_{\rm occ}[k] = -\frac{1}{2}p_0[1] + \frac{3}{2}p_0[1]p_{\rm occ}[0] + \left(p_0[1] + p_0[2]\right) p_{\rm occ}[1]. \tag{47}$$

The equality in Supplementary Equation (47) follows from the normalisation of $p_{\rm occ}[k]$, $2\sum_{k=2}^{+\infty} p_{\rm occ}[k] = 1 - p_{\rm occ}[0] - 2p_{\rm occ}[1]$.

The $p_{\rm occ}[k]$ weights in Supplementary Equation (47) are unknown, so we wish to bound them. Again using Equation (20), we find

$$h_3 - h_2 - p_0[4]p_{\rm occ}[2] = p_0[2]p_{\rm occ}[0] + \left(p_0[1] + p_0[3]\right) p_{\rm occ}[1] - \sum_{k=2}^{+\infty} \left(p_0[k-2] - p_0[k+2]\right) p_{\rm occ}[k]. \tag{48}$$

For $k \geq 2$ and $\rho^4 < (k+2)(k+1)k(k-1)$ and $k_{\rm min} = 2$ we have $p_0[k-2] - p_0[k+2] > 0$ such that

$$h_3 - h_2 - p_0[4]p_{\rm occ}[2] < p_0[2]p_{\rm occ}[0] + \left(p_0[1] + p_0[3]\right) p_{\rm occ}[1] < \frac{3}{2}p_0[1]p_{\rm occ}[0] + \left(p_0[1] + p_0[2]\right) p_{\rm occ}[1], \tag{49}$$

where the second inequality holds for $\rho^2 < 3$. Now using the transitive property of the inequality and the fact that $p_{\rm occ}[k] \leq 1$, Supplementary Equations (47) and (49) lead to Equation (23), a lower bound on h_2 for $\rho^2 < 3$ with a positive definite $p_{\rm occ}[k]$.

Bound on $p_{\rm tot}[n]$

We next turn to the bound on $p_{\rm tot}[n]$ stated in Equation (24), one that can be violated by a squeezed state in the cavity, as shown in Figure 5(a). Again, we bound the behaviour of $p_{\rm tot}[k]$ assuming only that $p_{\rm occ}[k]$ is positive definite. We start by using the fact that $p_{\rm tot}[n]$ is a convolution between $p_0[k]$ and $p_{\rm occ}[k]$,

$$p_{\rm tot}[n] = \sum_{k=0}^{+\infty} p_0[k]p_{\rm occ}[n-k] = p_0[0]p_{\rm occ}[n] + \frac{p_0[1]}{p_0[0]}p_0[0]p_{\rm occ}[n-1] + \sum_{k=2}^{+\infty} \frac{p_0[k]}{p_0[0]}p_0[0]p_{\rm occ}[n-k], \tag{50a}$$

$$p_{\rm tot}[n-1] - p_{\rm tot}[n-2] = p_0[0]p_{\rm occ}[n-1] + \sum_{k=2}^{+\infty} \left(p_0[k-1] - p_0[k-2]\right) p_{\rm occ}[n-k]. \tag{50b}$$

Substituting Supplementary Equation (50b) into Supplementary Equation (50a) we find

$$p_{\rm tot}[n] = p_0[0]p_{\rm occ}[n] + \frac{p_0[1]}{p_0[0]}\left[p_{\rm tot}[n-1] - p_{\rm tot}[n-2] + \sum_{k=2}^{+\infty}\left(\frac{p_0[0]p_0[k]}{p_0[1]} - p_0[k-1] + p_0[k-2]\right)p_{\rm occ}[n-k]\right]. \quad (51)$$

Now the term in braces in Supplementary Equation (51) is simply $e^{-\rho}k!\rho^{k-2}(k-\rho)(k-1)/k!$, which is greater than zero for $k \geq 2$ and $\rho < 2$. With $p_{\rm occ}[k]$ positive definite we then have Equation (24), a lower bound for $p_{\rm tot}[n]$ for $\rho < 2$.

Bound on height of fourth conductance plateau

Now we will derive a bound on the height of the fourth conductance plateau, a bound that is useful for inferring the non-classicality of a squeezed state directly from a transport measurement, as shown in Figure 5(b). The calculations described below follow from no more than Equations (19) and (20), and the assumption of a positive definite $p_{\rm occ}[k]$. This assumption shall be made throughout the calculation, but henceforth, will not be explicitly stated when it is employed.

1. *Height differences of conductance plateaus*

We start by expressing the height differences of the conductance plateaus in terms of the weights of the $P(E)$ functions. Using Equation (20) we can express the difference between the heights of the second and third conductance plateaus, as in Supplementary Equation (48),

$$h_3 - h_2 = p_0[2]p_{\rm occ}[0] + (p_0[1] + p_0[3])p_{\rm occ}[1] + p_0[4]p_{\rm occ}[2] + \sum_{k=3}^{+\infty}\left(p_0[k+2] - p_0[k-2]\right)p_{\rm occ}[k]. \quad (52)$$

Subtracting $\rho\left(2p_0[2] + p_0[4]\right)p_{\rm occ}[1]/2$ from both sides of Supplementary Equation (52) leads to

$$h_3 - h_2 - p_0[2]p_{\rm occ}[0] - \frac{\rho}{2}\left(2p_0[2] + p_0[4]\right)p_{\rm occ}[1] - p_0[4]p_{\rm occ}[2] + \sum_{k=3}^{+\infty}\left(p_0[k-2] - p_0[k+2]\right)p_{\rm occ}[k]$$
$$= \left[p_0[1] + p_0[3] - \frac{\rho}{2}\left(2p_0[2] + p_0[4]\right)\right]p_{\rm occ}[1]. \quad (53)$$

The right-hand-side is positive provided that $\rho < 1.525$, and consequently we have that the left-hand-side is greater than zero. Therefore, we can write

$$\sum_{k=3}^{+\infty}\left(p_0[k-2] - p_0[k+2]\right)p_{\rm occ}[k] > -h_3 + h_2 + p_0[2]p_{\rm occ}[0] + \frac{\rho}{2}\left(2p_0[2] + p_0[4]\right)p_{\rm occ}[1] + p_0[4]p_{\rm occ}[2]. \quad (54)$$

Again using Equation (20), we can also write an expression for the height difference of the third and fourth conductance plateaus,

$$\sum_{k=4}^{+\infty}\left(p_0[k-3] - p_0[k+3]\right)p_{\rm occ}[k] = -h_4 + h_3 + p_0[3]p_{\rm occ}[0] + (p_0[2] + p_0[4])p_{\rm occ}[1] + (p_0[1] + p_0[5])p_{\rm occ}[2]$$
$$+ p_0[6]p_{\rm occ}[3]. \quad (55)$$

Now we have $\rho\left(p_0[k-3] - p_0[k+3]\right)/2 > p_0[k-2] - p_0[k+2]$ for $\rho < 3.742$. This allows us to rewrite Supplementary Equation (55) as an inequality, and separating out the first term in the summation we have

$$\sum_{k=3}^{+\infty}\left(p_0[k-2] - p_0[k+2]\right)p_{\rm occ}[k] < \frac{\rho}{2}\left[p_0[3]p_{\rm occ}[0] + (p_0[2] + p_0[4])p_{\rm occ}[1] + (p_0[1] + p_0[5])p_{\rm occ}[2]\right.$$
$$\left. + p_0[6]p_{\rm occ}[3] - h_4 + h_3\right] + (p_0[1] - p_0[5])p_{\rm occ}[3]. \quad (56)$$

In Supplementary Equations (54) and (56) we have obtained an upper and lower bound on the sum of $p_{\text{occ}}[k]$. Applying the transitive property of the inequality between these equations allows us to eliminate this sum, leaving us with

$$\frac{\rho}{2}p_0[2]p_{\text{occ}}[1] < h_3 - h_2 - \frac{\rho}{2}(h_4 - h_3) + \left(\frac{\rho}{2}p_0[3] - p_0[2]\right)p_{\text{occ}}[0] + \left[\frac{\rho}{2}(p_0[1] + p_0[5]) - p_0[4]\right]p_{\text{occ}}[2] + \left(p_0[1] - p_0[5] + \frac{\rho}{2}p_0[6]\right)p_{\text{occ}}[3]. \tag{57}$$

Supplementary Equation (57) is now an inequality relating the second, third and fourth conductance plateaus.

Applying Equation (20) to the first and second conductance plateaus, as was done for Supplementary Equation (46) yields

$$p_0[2]p_{\text{occ}}[1] = h_2 - h_1 - p_0[1]p_{\text{occ}}[0] + (p_0[1] - p_0[3])p_{\text{occ}}[2] + (p_0[2] - p_0[4])p_{\text{occ}}[3] + \sum_{k=4}^{+\infty}(p_0[k-1] - p_0[k+1])\,p_{\text{occ}}[k]. \tag{58}$$

Dropping the summation in Supplementary Equation (58), on the grounds that the coefficients of $p_{\text{occ}}[k]$ are positive for $\rho < 4.472$, we are left with an inequality. Subsequently multiplying both sides of the inequality by $\rho/2$ we find

$$\frac{\rho}{2}p_0[2]p_{\text{occ}}[1] > \frac{\rho}{2}(h_2 - h_1) - \frac{\rho}{2}p_0[1]p_{\text{occ}}[0] + \frac{\rho}{2}\left(p_0[1] - p_0[3]\right)p_{\text{occ}}[2] + \frac{\rho}{2}\left(p_0[2] - p_0[4]\right)p_{\text{occ}}[3]. \tag{59}$$

Considering Supplementary Equations (57) and (59), we see that we have an upper and a lower bound on $p_{\text{occ}}[1]$. Using the transitive property of the inequality we find

$$h_3 - h_2 - \frac{\rho}{2}(h_4 - h_3 + h_2 - h_1) > -\frac{\rho}{2}p_0[3]p_{\text{occ}}[0] + \left[p_0[4] - \frac{\rho}{2}(p_0[3] + p_0[5])\right]p_{\text{occ}}[2] + \left[-p_0[1] + p_0[5] + \frac{\rho}{2}\left(p_0[2] - p_0[4] - p_0[6]\right)\right]p_{\text{occ}}[3]. \tag{60}$$

We have now obtained a lower bound on a linear combination of heights of the first four conductance plateaus. However, the bound still contains the (unknown) $p_{\text{occ}}[k]$ ($k = 0, 2, 3$). Therefore, we now seek to bound these quantities in terms of the known $p_0[k]$ and the measurable h_k ($k = 1, 2, 3, 4$).

2. *Bounding* $p_{\text{occ}}[0]$

Using Equation (19) with the normalisation conditions $1 = \sum_{n=-\infty}^{+\infty}\sum_{m=0}^{+\infty}p_0[m]p_{\text{occ}}[n-m]$ and $1 = \sum_{m=0}^{+\infty}p_0[m]$, we can show that

$$1 - h_1 = \sum_{n=1}^{+\infty}\sum_{m=0}^{+\infty}p_0[m]\left(p_{\text{occ}}[n-m] - p_{\text{occ}}[n+m]\right) \tag{61a}$$

$$= (1 - p_0[0])p_{\text{occ}}[0] + 2\sum_{k=1}^{+\infty}\left(1 - \sum_{m=0}^{k-1}p_0[m] - p_0[k]/2\right)p_{\text{occ}}[k]. \tag{61b}$$

Truncating the summation on the right-hand-side of Supplementary Equation (61b) at the $p_{\text{occ}}[3]$ term and rearranging leads to a bound on $p_{\text{occ}}[0]$,

$$p_{\text{occ}}[0] < \frac{1 - h_1}{1 - p_0[0]} - 2\frac{1 - p_0[0] - p_0[1]/2}{1 - p_0[0]}p_{\text{occ}}[1] - 2\frac{1 - p_0[0] - p_0[1] - p_0[2]/2}{1 - p_0[0]}p_{\text{occ}}[2] - 2\frac{1 - p_0[0] - p_0[1] - p_0[2] - p_0[3]/2}{1 - p_0[0]}p_{\text{occ}}[3]. \tag{62}$$

Using the bound of Supplementary Equation (62) in Supplementary Equation (60) leads to a coefficient on the $p_{\text{occ}}[2]$ term (on the smaller side of the inequality), $\rho p_0[3](1 - p_0[0] - p_0[1] - p_0[2]/2)/(1 - p_0[0]) + p_0[4] - \rho\left(p_0[3] + p_0[5]\right)/2$. This coefficient is positive for $1.011 < \rho < 5.235$ and so the $p_{\text{occ}}[2]$ term may be removed from the inequality. This leaves us with

$$h_3 - h_2 - \frac{\rho}{2}\left(h_4 - h_3 + h_2 - h_1\right) > -\frac{\rho}{2}p_0[3]\frac{1 - h_1}{1 - p_0[0]} + \rho p_0[3]\frac{1 - p_0[0] - p_0[1]/2}{1 - p_0[0]}p_{\text{occ}}[1] + \rho p_0[3]\frac{1 - p_0[0] - p_0[1] - p_0[2] - p_0[3]/2}{1 - p_0[0]}p_{\text{occ}}[3] + \left[\frac{\rho}{2}\left(p_0[2] - p_0[4] - p_0[6]\right) - p_0[1] + p_0[5]\right]p_{\text{occ}}[3]. \tag{63}$$

Comparing Supplementary Equation (63) to Supplementary Equation (60) we have one fewer unknown $p_{\rm occ}[k]$ in the bound; we now seek to reduce this number further.

3. Bounding $p_{\rm occ}[3]$

Applying Equations (19) and (20) iteratively we find the expressions for the height of the fourth conductance plateau,

$$h_4 = p_{\rm tot}[0] + \sum_{k=1}^{3} (p_{\rm tot}[k] + p_{\rm tot}[-k]) + 2\sum_{k=4}^{+\infty} p_{\rm tot}[-k] \tag{64a}$$

$$= \sum_{l=0}^{+\infty} p_0[l] p_{\rm occ}[l] + \sum_{k=1}^{3}\sum_{l=k}^{+\infty} p_0[l-k] p_{\rm occ}[l] + \sum_{k=1}^{3}\sum_{l=-k}^{+\infty} p_0[l+k] p_{\rm occ}[l] + 2\sum_{k=4}^{+\infty}\sum_{l=k}^{+\infty} p_0[l-k] p_{\rm occ}[l]. \tag{64b}$$

The second line follows from expanding the total $P(E)$ weights as convolutions of the vacuum and occupied $P(E)$ weights. Next we can write an expression for $1-h_4$ using Supplementary Equation (64b) and the normalisation condition $1 = \sum_{m=0}^{+\infty} p_0[m] p_{\rm occ}[0] + 2\sum_{m=0}^{+\infty} p_0[m] \sum_{k=1}^{+\infty} p_{\rm occ}[k]$. Truncating the resulting expression at the $p_{\rm occ}[4]$ and $p_0[4]$ terms leads us to the inequality

$$\begin{aligned} 1-h_4 > {} & (1-p_0[0]-p_0[1]-p_0[2]-p_0[3])\, p_{\rm occ}[0] + (1-p_0[0]-p_0[1]-p_0[2])\, p_{\rm occ}[1] \\ & + (1-p_0[0]-p_0[1])\, p_{\rm occ}[2] + (1-p_0[0])\, p_{\rm occ}[3] + (1-p_0[0])\, p_{\rm occ}[4]. \end{aligned} \tag{65}$$

Writing Supplementary Equation (55) as a lower bound for $h_3 - h_4$ by removing the summation, and adding the resulting inequality to that of Supplementary Equation (65), we find

$$\begin{aligned} 1 + h_3 - 2h_4 > {} & (1-p_0[0]-p_0[6]) p_{\rm occ}[3] + (1-p_0[0]-2p_0[1]-p_0[5]) p_{\rm occ}[2] \\ & + (1-p_0[0]-p_0[1]-2p_0[2]-p_0[4]) p_{\rm occ}[1] + (1-p_0[0]-p_0[1]-p_0[2]-2p_0[3]) p_{\rm occ}[0]. \end{aligned} \tag{66}$$

The coefficient of $p_{\rm occ}[2]$ is positive for $\rho > 1.274$, and so we may remove it from this inequality. Further, $p_{\rm occ}[0] < (1-h_1)/(1-p_0[0])$, which follows from truncating Supplementary Equation (61b) at the first term. Using this bound, Supplementary Equation (66) can be written as a bound on $p_{\rm occ}[3]$,

$$\begin{aligned} p_{\rm occ}[3](1-p_0[0]-p_0[6]) < {} & 1 + h_3 - 2h_4 + (p_0[0]+p_0[1]+p_0[2]+2p_0[3]-1)\frac{1-h_1}{1-p_0[0]} \\ & + (p_0[0]+p_0[1]+2p_0[2]+p_0[4]-1) p_{\rm occ}[1]. \end{aligned} \tag{67}$$

4. Final bounds on fourth conductance plateau

Before stating our final bounds on the conductance plateaus we introduce some short-hand notation for the complicated functions of ρ that arise:

$$\mathcal{A} \equiv \frac{-\rho(p_0[2]-p_0[4]+p_0[6])/2 + p_0[1] - p_0[5]}{1-p_0[0]-p_0[6]} - \rho\frac{p_0[3]}{1-p_0[0]}\frac{1-p_0[0]-p_0[1]-p_0[2]-p_0[3]/2}{1-p_0[0]-p_0[6]}, \tag{68a}$$

$$\mathcal{B} \equiv 1/(1-p_0[0]), \tag{68b}$$

$$\mathcal{C} \equiv p_0[0]+p_0[1]+p_0[2]+2p_0[3]-1. \tag{68c}$$

Note that $\mathcal{A} > 0$ for $\rho < 1.859$, $\mathcal{C} > 0$ for $\rho < 2.318$, and $\mathcal{B}$ is unconditionally positive.

Now using the bound of Supplementary Equation (67) in Supplementary Equation (63) we see that the coefficient of $p_{\rm occ}[1]$ (appearing on the smaller side of the inequality), $\rho\mathcal{B}p_0[3](1-p_0[0]-p_0[1]/2)+\mathcal{A}(1-p_0[0]-p_0[1]-2p_0[2]-p_0[4])$, is positive for $1.042 < \rho < 5.212$, and therefore the $p_{\rm occ}[1]$ term may be discarded. This leaves us with an inequality which may be expressed as an upper bound on the height of the fourth conductance plateau,

$$h_4(2\mathcal{A}+\rho/2) < \mathcal{A}(1+\mathcal{BC}) + \mathcal{B}\rho p_0[3]/2 - [\mathcal{ABC} - \rho(1-\mathcal{B}p_0[3])/2]\, h_1 - (1+\rho/2)h_2 + (1+\rho/2+\mathcal{A})h_3. \tag{69}$$

This bound is valid for $1.274 < \rho < 1.525$. We stress that these bounds are expressed in terms of the known $p_0[k]$ and the measurable h_k $(k = 1, 2, 3)$. The bounds apply for a classical distribution in the coupled electromagnetic mode, and may be violated in the presence of a non-classical field.

Supplementary References

[1] G.-L. Ingold & Y. Nazarov In "Single Charge tunnelling", edited by H. Grabert and M. H. Devoret, *NATO ASI Series B*, Vol. **294**, (Plenum Press, New York, 1992).

[2] E. Zakka-Bajjani, J. Segala, F. Portier, P. Roche, D. C. Glattli, A. Cavanna & Y. Jin Experimental Test of the High-Frequency Quantum Shot Noise Theory in a Quantum Point Contact. *Phys. Rev. Lett.* **99**, 236803 (2007).

[3] J. Gabelli & B. Reulet Dynamics of Quantum Noise in a Tunnel Junction under ac Excitation. *Phys. Rev. Lett.* **100**, 026601 (2008).

[4] E. Zakka-Bajjani, J. Dufouleur, N. Coulombel, P. Roche, D. C. Glattli & F. Portier Experimental Determination of the Statistics of Photons Emitted by a Tunnel Junction. *Phys. Rev. Lett.* **104**, 206802 (2010).

[5] G. Gasse, L. Spietz, C. Lupien & B. Reulet Observation of quantum oscillations in the photoassisted shot noise of a tunnel junction. *Phys. Rev. B* **88**, 241402(R) (2013).

[6] J.-R. Souquet, I. Safi & P. Simon Dynamical Coulomb blockade in an interacting one-dimensional system coupled to an arbitrary environment. *Phys. Rev. B* **88**, 205419 (2013).
